# Runx2 is required for the proliferation of osteoblast progenitors and induces proliferation by regulating *Fgfr2* and *Fgfr3*

**DOI:** 10.1038/s41598-018-31853-0

**Published:** 2018-09-10

**Authors:** Tetsuya Kawane, Xin Qin, Qing Jiang, Toshihiro Miyazaki, Hisato Komori, Carolina Andrea Yoshida, Viviane Keiko dos Santos Matsuura-Kawata, Chiharu Sakane, Yuki Matsuo, Kazuhiro Nagai, Takafumi Maeno, Yuki Date, Riko Nishimura, Toshihisa Komori

**Affiliations:** 1 Department of Cell Biology, Nagasaki University Graduate School of Biomedical Sciences, Nagasaki, 852-8588 Japan; 2 Basic and Translational Research Center for Hard Tissue Disease, Nagasaki University Graduate School of Biomedical Sciences, Nagasaki, 852-8588 Japan; 3 Transfusion and Cell Therapy Unit, Nagasaki University Hospital, Nagasaki, 852-8501 Japan; 4 Department of Orthopedic Surgery, Osaka City University Graduate School of Medicine, Osaka, 545-8585 Japan; 5 Department of Molecular Bone Biology, Nagasaki University Graduate School of Biomedical Sciences, Nagasaki, 852-8588 Japan; 6 Department of Molecular and Cellular Biochemistry, Osaka University Graduate School of Dentistry, Osaka, 565-0871 Japan

## Abstract

Runx2 and Sp7 are essential transcription factors for osteoblast differentiation. However, the molecular mechanisms responsible for the proliferation of osteoblast progenitors remain unclear. The early onset of *Runx2* expression caused limb defects through the *Fgfr1*–3 regulation by Runx2. To investigate the physiological role of Runx2 in the regulation of *Fgfr1*–*3*, we compared osteoblast progenitors in *Sp7*^−/−^ and *Runx2*^−/−^ mice. Osteoblast progenitors accumulated and actively proliferated in calvariae and mandibles of *Sp7*^−/−^ but not of *Runx2*^−/−^ mice, and the number of osteoblast progenitors and their proliferation were dependent on the gene dosage of *Runx2* in *Sp7*^−/−^ background. The expression of *Fgfr2* and *Fgfr3*, which were responsible for the proliferation of osteoblast progenitors, was severely reduced in *Runx2*^−/−^ but not in *Sp7*^−/−^ calvariae. Runx2 directly regulated *Fgfr2* and *Fgfr3*, increased the proliferation of osteoblast progenitors, and augmented the FGF2-induced proliferation. The proliferation of *Sp7*^−/−^ osteoblast progenitors was enhanced and strongly augmented by FGF2, and *Runx2* knockdown reduced the FGF2-induced proliferation. Fgfr inhibitor AZD4547 abrogated all of the enhanced proliferation. These results indicate that Runx2 is required for the proliferation of osteoblast progenitors and induces proliferation, at least partly, by regulating *Fgfr2* and *Fgfr3* expression.

## Introduction

Osteoblast differentiation is regulated by Runx2, Sp7, and canonical Wnt signaling^1^. Since *Runx2* is expressed in *Sp7*-deficient (*Sp7*^−/−^) mice and *Ctnnb1* conditional knockout mice, Runx2 is the furthest upstream transcription factor in the regulation of osteoblast differentiation, and Runx2, Sp7, and canonical Wnt signaling are mutually regulated and maintain their expression^1,2^. Both osteoblast differentiation and the expansion of osteoblast progenitors are essential for bone development and bone regeneration. Although the mechanism for osteoblast differentiation has been well studied, the mechanism for the proliferation of osteoblast progenitors remains to be clarified^1^.

Many *in vitro* studies of Runx2 in the proliferation of osteoblastic cells has been reported. The proliferation of *Runx2*^−/−^ calvarial cells was greater than that of wild-type calvarial cells; Runx2 induces G1 cell-cycle arrest through the induction of p27^KIP1^ in osteosarcoma cells; Runx2 expression is up-regulated in the cessation of cell proliferation and down-regulated to minimal levels during the early S phase and mitosis in MC3T3-E1 preosteoblastic cells; Runx2 suppresses the proliferation of cells with osteogenic potential and osteosarcoma cells, and the introduction of siRNA against *Runx2* into human mesenchymal stem cells increases proliferation^3–7^. Thus, all previous reports show that Runx2 inhibits the proliferation of osteoblastic cells *in vitro*.

Four fibroblast growth factor receptor (Fgfr) genes have been identified in mammals (*Fgfr1* to *Fgfr4*). The affinity and specificity of Fgfr1–3 are regulated by tissue-specific alternative splicing, which occurs in the region encoding the carboxyl-terminal half of Ig domain III creating the different isoforms, IIIb and IIIc^8^. *Fgf2*, *Fgf4*, *Fgf9*, *Fgf18*, *Fgfr1*, *Fgfr2*, and *Fgfr3* are expressed in mesenchymal cells in the calvaria^9–16^. The importance of FGF signaling in human skull development has been revealed by the identification of gain-of-function mutations in the *FGFR1*, *FGFR2*, and *FGFR3* genes in a number of craniosynostosis syndromes, such as Apert, Crouzon, Pfeiffer, and Muenke syndromes and Thanatophoric dysplasia^17^. Fgf10, Fgfr1c, and Fgfr2c, which are expressed in mesenchyme, and Fgf8, Fgf4, and Fgfr2b, which are expressed in ectoderm, form an epithelial-mesenchymal interaction loop during the proximodistal and anteroposterior patterning of the limb bud^18,19^, and craniosynostosis is accompanied by limb defects in Apert and Pfeiffer syndromes^17^.

FGF signals trigger a number of responses in target cells, including stemness, proliferation, anti-apoptosis, drug resistance, angiogenesis, epithelial-to-mesenchymal transition, and invasion, through RAS-MAPK, PI3K-AKT, PLCγ and DAG, and PLCγ and IP3. Furthermore, the FGF signaling pathway has crosstalk with the canonical Wnt signaling cascade. In cell proliferation, FGF signaling plays important roles through RAS-MAPK, PI3K-AKT, and canonical Wnt signaling^20,21^. Genomic alterations in FGFRs are associated with various cancers, including breast cancer, lung cancer, gastric cancer, multiple myeloma, myeloproliferative syndrome, rhabdomyosarcoma, peripheral T-cell lymphoma, uterine tumors, and bladder tumors^20,21^.

We previously reported that *Runx2* transgenic mice under the control of the *Prrx1* promoter, which directs transgene expression to the limb bud mesenchyme and cranial mesenchyme from embryonic day (E) 9.5^22^, exhibit craniosynostosis, ectopic bone formation, and limb defects^23^. Since FGF signaling plays an important role in limb development, we examined the involvement of Runx2 in the FGF signaling pathway in this study. Runx2 directly regulated the *Fgfr1*, *Fgfr2*, and *Fgfr3* genes. In order to elucidate the physiological role of Runx2 in the regulation of *Fgfr1*–*3* expression, we further examined the proliferation of osteoblast progenitors. We found that Runx2 was required for the proliferation of osteoblast progenitors, and also that it induced proliferation, at least in part, through the regulation of *Fgfr2* and *Fgfr3*.

## Results

### Defects in Fgf signaling for limb development in Tg(*Prrx1-Runx2*) mice

**Figure 1:**
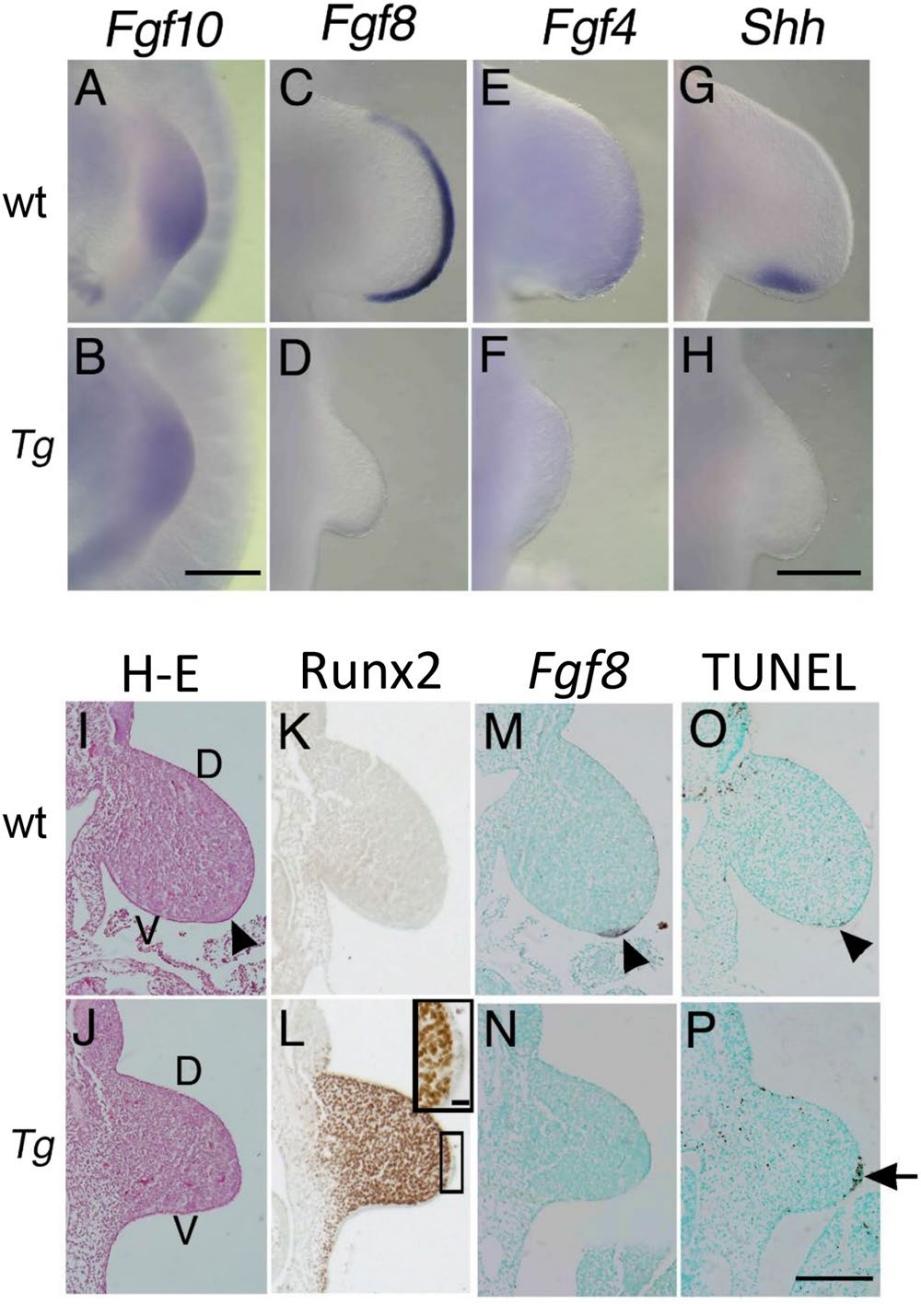
Limb development in *Tg(Prrx1-EGFP-Runx2)* mice (**A–H**) Whole mount *in situ* hybridization. Whole mount *in situ* hybridization of the forelimb buds of wild-type mice (wt) (**A**,**C**,**E**,**G**) and *Tg(Prrx1-EGFP-Runx2)* mice with strong expression (*Tg*) (**B**,**D**,**F**,**H**) at E10.0 (**A**,**B**) and E10.5 (**C**–**H**) using *Fgf10* (**A**,**B**), *Fgf8* (**C**,**D**), *Fgf4* (**E**,**F**), and *Shh* (**G,H**) probes. (**I**–**P**) Histological analysis. A histological analysis of the forelimb buds of a wild-type mouse (wt) (**I**,**K**,**M**,**O**) and *Tg(Prrx1-EGFP-Runx2)* (*Tg*) mouse with strong expression (*Tg*) (**J,L,N,P**) at E10.5. (**I,J**) H-E staining. The AER is observed in the wild-type mouse (I, arrowhead), but not in the *Tg(Prrx1-EGFP-Runx2)* mouse (**J**). D, dorsal; V, ventral. (**K,L**) Immunohistochemical analysis of Runx2 protein expression. The boxed region in L is amplified in the window. (**M,N**) *In situ* hybridization using the *Fgf8* probe. (**O**,**P**) TUNEL staining. F_0_ littermates of wild-type and *Tg(Prrx1-EGFP-Runx2)* mice were compared in each whole mount *in situ* hybridization and histological analysis. Scale bars: 20 μm (**A**,**B**); 200 μm (**C–H**); 200 μm (**I–P**); 20 μm (inset in **L**).

We previously reported that the early onset of *Runx2* expression causes craniosynostosis, ectopic bone formation, and limb defects, and also that the severity of limb defects depends on the expression levels of the transgene^23^. An epithelial-mesenchymal interaction loop formed by Fgfs and Fgfrs is essential for limb development. Fgf10, which first appears in the mesenchyme, has affinity for Fgfr2b in the apical ectodermal ridge (AER), which is a thickening of the ectoderm at the apex of the developing limb bud and is formed along the border of dorsal and ventral ectoderm, and induces Fgf8 and Fgf4 in the AER. Fgf8 and Fgf4 have affinity for Fgfr1c and Fgfr2c expressed in the mesenchyme, and promote mesenchymal proliferation and the outgrowth of limb buds^10,24–32^. The Fgf8 and Fgf4, which are expressed in AER, and Shh, which is expressed in the zone of polarizing activity (ZPA), mutually support the expression^33–35^. In order to elucidate the mechanisms responsible for limb defects, we examined the expression of *Fgf10* at E10.0 and that of *Fgf8*, *Fgf4*, and *Shh* at E10.5 in *Tg(Prrx1-EGFP-Runx2*) mice with high expression levels using whole mount *in situ* hybridization (Fig. 1A–H). Since *Tg(Prrx1-EGFP-Runx2)* mice were lethal at birth, we analyzed F_0_ littermates of wild-type and *Tg(Prrx1-EGFP-Runx2)* mice in each whole mount *in situ* hybridization. Therefore, the severity of the defect in limb development was different among F_0_ *Tg(Prrx1-EGFP-Runx2)* mice depending on the expression level of the transgene as previously described^23^. *Fgf10* mRNA was detected in wild-type and *Tg(Prrx1-EGFP-Runx2)* mice, while *Fgf8*, *Fgf4*, and *Shh* mRNA was detected in wild-type mice, but not in *Tg(Prrx1-EGFP-Runx2)* mice. In histological analyses, the AER was observed in the limb buds of wild-type mice, but was not apparent in *Tg(Prrx1-EGFP-Runx2)* mice at E10.5 (Fig. 1I,J). The endogenous Runx2 protein was undetectable in wild-type mice, while the Runx2 protein was present in mesenchymal cells, but not in the epithelium of the limb buds of *Tg(Prrx1-EGFP-Runx2)* mice (Fig. 1K,L). *Fgf8* mRNA was detected in the AER of the limb buds of wild-type mice, but not in *Tg(Prrx1-EGFP-Runx2)* mice (Fig. 1M,N), and the number of TUNEL-positive cells was higher in the presumptive AER region of *Tg(Prrx1-EGFP-Runx2)* mice than in the AER of the limbs of wild-type mice (Fig. 1O,P). Enhanced apoptosis in the AER region was also observed in *Fgf4* and *Fgf8* double mutant mice^32^. Therefore, these results suggest that FGF10, which was expressed in mesenchymal cells, failed to induce *Fgf8* and *Fgf4* mRNA expression in the ectoderm, leading to the apoptosis of cells in the AER region of *Tg(Prrx1-EGFP -Runx2)* mice with high expression levels. Thus, ectopic expression of *Runx2* in limb bud mesenchyme disturbed the induction of *Fgf8* and *Fgf4* expression in ectoderm by Fgf10 produced in mesenchyme.

### Runx2 regulates the expression of *Fgfr1, Fgfr2,* and *Fgfr3*

**Figure 2:**
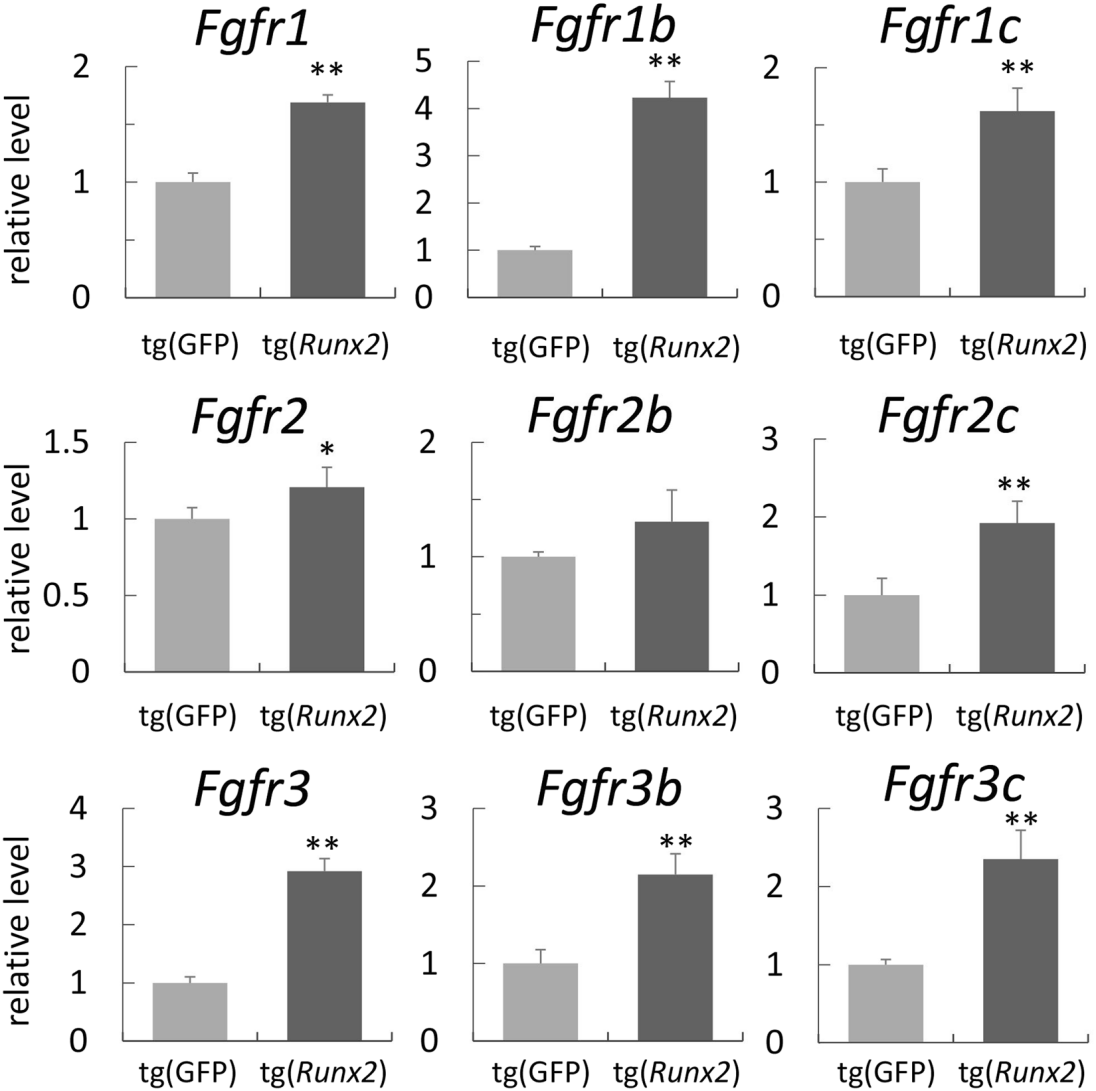
Real-time RT-PCR analyses of the expression of *Fgfr*1, *Fgfr2*, and *Fgfr3* The expression of *Fgfr*1, *Fgfr2*, and *Fgfr3* and their respective IIIb and IIIc isoform mRNA in *Tg(Prrx1-EGFP)* and *Tg(Prrx1-EGFP-Runx2)* mice was measured by real-time RT-PCR in triplicate. EGFP-positive cells were collected from limb buds of more than 70 F_0_ EGFP-positive embryos each in *Tg(Prrx1-EGFP-Runx2)* mice and *Tg(Prrx1-EGFP)* mice at E10.5 by sorting EGFP-positive cells using FACS, the EGFP-positive cells obtained in each sorting were pooled, and mRNA was extracted from the pooled cells. We normalized values to that of *Gapdh*. Values in wild-type mice were defined as 1, and the relative levels are shown. Data are the mean ± SD. *p < 0.05, **p < 0.01.

**Figure 3:**
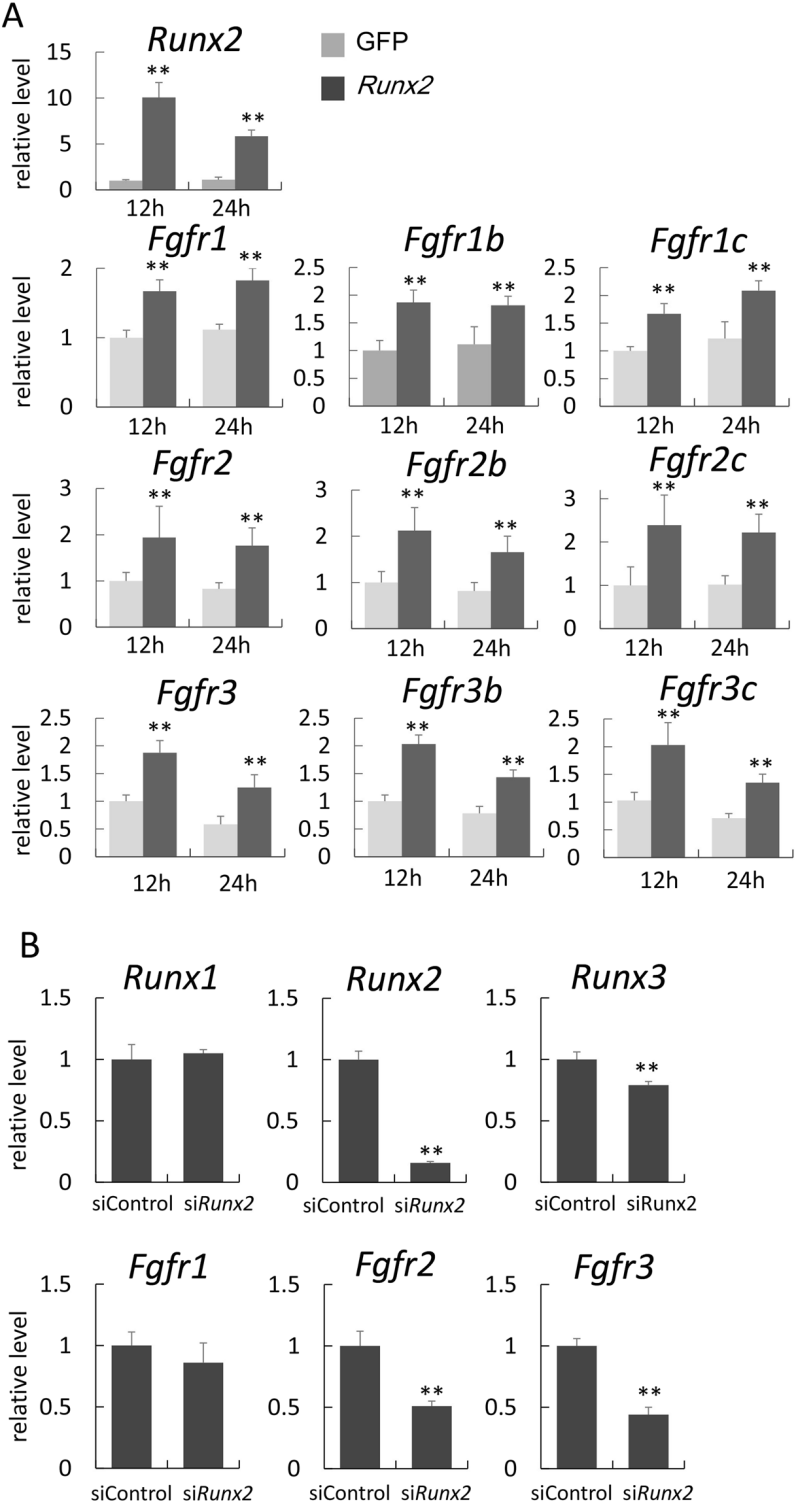
(**A**) Induction of *Fgfr*1, *Fgfr2*, and *Fgfr3* by *Runx2 in vitro*. RNA was extracted from wild-type osteoblast progenitors that had been infected with an adenovirus expressing type II *Runx2* and *EGFP* or *EGFP* alone. Samples were harvested 12 and 24 hrs after infection. Values in cells infected with the *EGFP-*expressing adenovirus were defined as 1, and the relative levels are shown. Data are the mean ± SD of 4 wells. **p < 0.01. (**B**) Suppression of *Fgfr*1, *Fgfr2*, and *Fgfr3* expression by *Runx2* siRNA. Osteoblast progenitors were prepared from the calvariae of wild-type newborn mice, and transfected with *Runx2* siRNA. RNA was extracted 48 hours after transfection, and real-time RT-PCR was performed. Values in siRNA for the control were defined as 1, and the relative levels are shown. Data are the mean ± SE of 3 wells. **p < 0.01. Similar results were obtained in three independent experiments and representative data are shown.

Since *Fgf10* mRNA was detected in mesenchymal cells, whereas *Fgf8* and *Fgf4* mRNA was not observed in the epithelium in *Tg(Prrx1-EGFP-Runx2)* mice, the expression of Fgfrs or their isoforms might have been disturbed in *Tg(Prrx1-EGFP-Runx2)* mice. Therefore, we examined the expression of *Fgfr1*–*3* and their isoforms by real-time RT-PCR using RNA from EGFP-positive cells sorted from the cell suspensions of *Tg(Prrx1-*EGFP*)* mice and *Tg(Prrx1-EGFP-Runx2)* mice at E10.5 (Fig. 2). *Fgfr1b*, *Fgfr1c*, *Fgfr2c*, *Fgfr3b*, and *Fgfr3c* mRNA levels were significantly higher in *Tg(Prrx1-EGFP-Runx2)* mice than in *Tg(Prrx1*-*EGFP)* mice. The *Runx2* expression in *Tg(Prrx1-EGFP-Runx2)* mice was 16 times higher than that in *Tg(Prrx1-EGFP)* mice in the microarray analysis using the sorted EGFP-positive cells (data not shown). Since Runx2 expression was not detected in wild-type limb buds (Fig. 1K), the regulation of Fgfrs at this developmental stage is not a physiological function of Runx2. Therefore, we examined whether Runx2 induces the expression of *Fgfr1*–*3* in osteoblast progenitors prepared from wild-type calvarial cells as described in the Materials and methods (Fig. 3A). Infection with type II *Runx2*-expressing adenovirus induced *Fgfr1*, *Fgfr2*, and *Fgfr3* mRNA and their IIIb and IIIc isoform mRNA. Since the induction of *Fgfr2* by Runx2 in osteoblast progenitors was more apparent than that in limb bud mesenchymal cells at E10.5 (Figs 2 and 3A), it is likely that the transcription factors and/or co-factors collaborating with Runx2 for *Fgfr2* expression is more abundant in osteoblast progenitors than limb bud mesenchymal cells at E10.5. Further, the expression of the molecules related to alternative splicing may be also different, because *Fgfr2b* expression was not significantly upregulated in limb bud mesenchymal cells at E10.5 (Fig. 2). The induction of *Fgfr1*, *Fgfr2*, *Fgfr3*, and *Fgfr4* mRNA by Runx2 has also been reported by microarray analysis using RNA from immortalized *Runx2*^−/−^ calvarial cells infected with *Runx2*-expressing adenovirus^36^. The introduction of *Runx2* siRNA in osteoblast progenitors reduced the expression of *Fgfr2* and *Fgfr3* (Fig. 3B). However, *Runx2* siRNA reduced *Runx3* mRNA as well as *Runx2* mRNA, although the siRNA sequence was specific for *Runx2*. Therefore, we examined whether Runx2 regulates *Runx3* expression (Supplemental Fig. 1). *Runx3*, but not *Runx1* expression was markedly weaker in *Runx2*^−/−^ calvariae than in wild-type and *Sp7*^−/−^ calvariae (Supplemental Fig. 1A). The overexpression of *Runx2* induced the expression of *Runx3*, but not that of *Runx1* in wild-type osteoblast progenitors (Supplemental Fig. 1B). The overexpression of *Runx3* induced neither *Runx1* nor *Runx2*. Furthermore, the overexpression of *Runx3* failed to induce *Fgfr1*, *Fgfr2*, and *Fgfr3* (Supplemental Fig. 1B). These results indicated that Runx2, but not Runx3 regulates *Fgfr1*, *Fgfr2*, and *Fgfr3*.

### Direct regulation of promoters of *Fgfr1, Fgfr2,* and *Fgfr3* by Runx2

**Figure 4:**
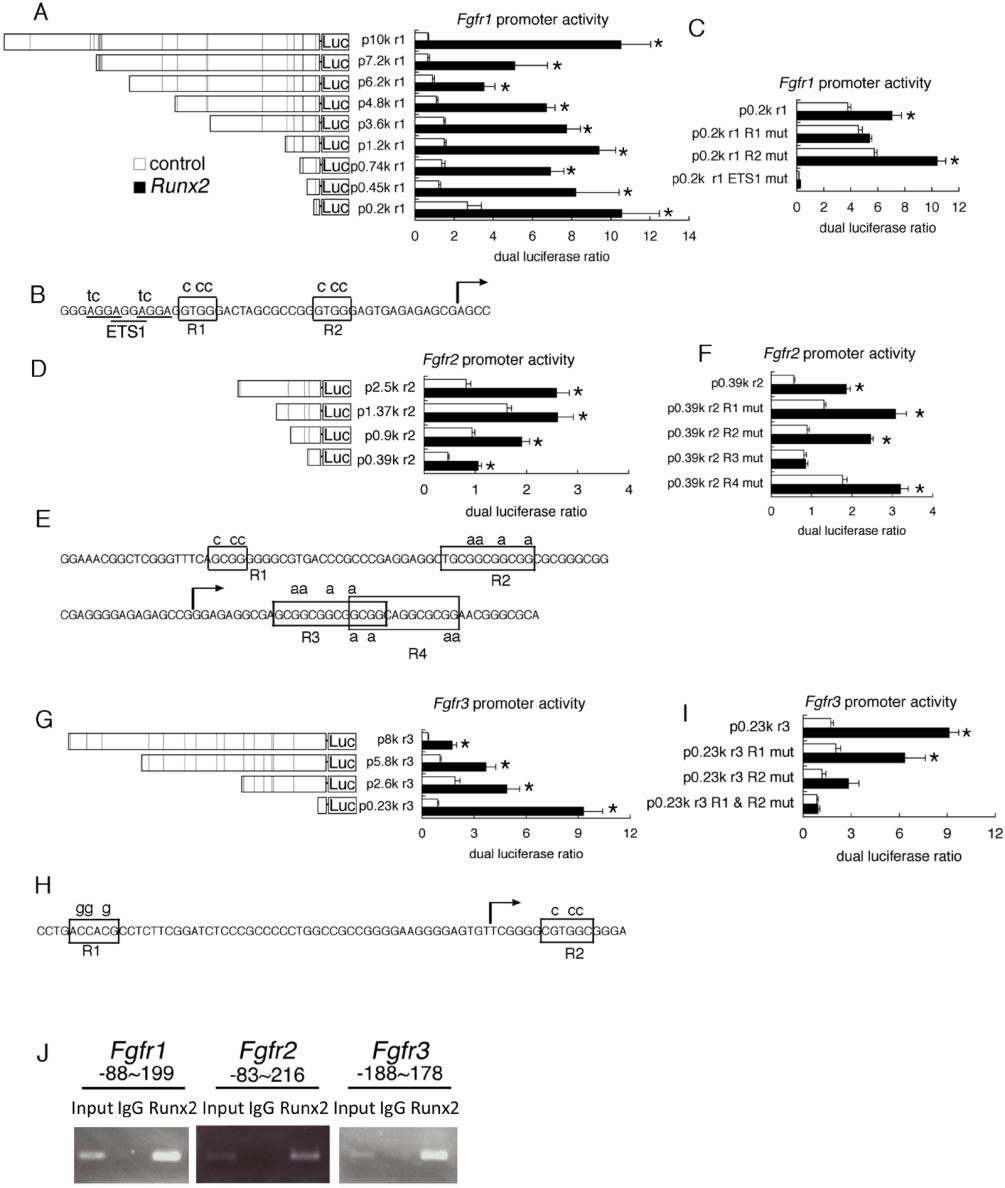
Reporter and ChIP assays of *Fgfr1*, *Fgfr2*, and *Fgfr3* promoters. (**A–C**) Reporter assays of the *Fgfr1* promoter. (**A**) Schematic diagrams of the reporter vectors of the *Fgfr1* promoter and their luciferase (Luc) activities. (**B**) The nucleotide sequence containing putative Runx2-binding sites in the 0.2-kb fragment. Ets1-binding sites are underlined and putative Runx2-binding sites (R1 and R2) are boxed. The mutated sequences are shown above the boxes and lines. (**C**) Reporter activities of the 0.2-kb construct (p0.2k r1) and the 0.2-kb constructs carrying a mutated R1, R2, or Ets1 site. (**D**–**F**) Reporter assays of the *Fgfr2* promoter. (**D**) Schematic diagrams of the reporter vectors of the *Fgfr2* promoter and their luciferase activities. (**E**) The nucleotide sequence containing putative Runx2-binding sites in the 0.39-kb fragment. The putative Runx2-binding sites (R1–4) are boxed, and the mutated sequences are shown above or below the boxes. (**F**) Reporter activities of the 0.39-kb construct (p0.39k r2) and the 0.39-kb constructs carrying mutated R1, R2, R3, or R4. (**G**–**I**) Reporter assays of the *Fgfr3* promoter. (**G**) Schematic diagrams of the reporter vectors of the *Fgfr3* promoter and their luciferase activities. (**H**) The nucleotide sequence containing putative Runx2-binding sites in the 0.23-kb fragment. The putative Runx2-binding sites (R1, R2) are boxed, and the mutated sequences are shown above the boxes. (**I**) Reporter activities of the 0.23-kb construct (p0.23k r3) and the 0.23-kb constructs carrying mutated R1, R2, or R1 and R2. Vertical lines in the diagrams represent the positions of consensus Runx2-binding motifs (**A**,**D**,**G**), and arrows indicate reported transcription start sites (**B,E,H**). In all reporter assays, C3H10T1/2 cells were transfected with an empty (open column) or *Runx2*-expressing (closed column) vector. Data are the mean ± SD of 4 wells. *p < 0.01 versus the control. Three independent experiments were performed and representative data are shown. (**J**) ChIP assays. DNA before (input) and after immunoprecipitation with a monoclonal anti-Runx2 antibody (Runx2) or mouse IgG (IgG) was amplified by PCR using primers that amplify the sequences of *Fgfr1* (−88 ~+199), *Fgfr2* (−83 ~+216), and *Fgfr3* (−188 ~+178). Similar results were obtained in three independent experiments and representative data are shown.

Since Runx2 induced the expression of *Fgfr1*, *Fgfr2*, and *Fgfr3 in vivo* and *in vitro*, we performed reporter assays using the promoter regions of *Fgfr1*, *Fgfr2*, and *Fgfr3* (Fig. 4). In the reporter assay using a 10-kb fragment of the *Fgfr1* promoter region, which contains eighteen consensus Runx2-binding motifs (TGPyGGPy), Runx2 strongly induced reporter activity (Fig. 4A). Serial deletions of the 10-kb fragment showed that the distal 4 kb is, in part, responsible for Runx2-dependent transcriptional activation; however, further deletions augmented Runx2-dependent transcriptional activation, and Runx2 still activated the reporter activity of the 0.2-kb fragment. In the 0.2-kb fragment, there was one overlapping Ets1-binding site and two putative Runx-binding sites, which contained the core four nucleotides of the consensus Runx2-binding motifs, R1 and R2 (Fig. 4B). The mutation of R1, but not R2 completely abolished Runx2-dependent transcriptional activation (Fig. 4C). Since R1 and the Ets1-binding sites are closely located, Runx2 and Ets1 may co-operatively bind to and activate the *Fgfr1* promoter, as previously described for the *Spp1* promoter^37^. It was not possible to confirm this because the mutation of the Ets1-binding site completely abolished the basal activity of the promoter (Fig. 4C).

In the reporter assay using a 2.5-kb fragment of the *Fgfr2* promoter region, which contains four consensus Runx2-binding sites, Runx2 induced reporter activity (Fig. 4D). Serial deletions of the 2.5-kb fragment showed that the distal 1.13 kb was, in part, responsible for Runx2-dependent transcriptional activation; however, Runx2 maintained the ability to enhance the transcription of the reporter vector containing a 0.39-kb deletion fragment (Fig. 4D). The 0.39-kb fragment contained one consensus Runx2-binding motif (R2) and three putative Runx2-binding sites consisting of the four core nucleotides of the consensus Runx2-binding sequences (R1, R3, R4) (Fig. 4E). The mutation of R3 abolished Runx2-dependent transcriptional activation (Fig. 4F).

In the reporter assay using an 8-kb fragment of the *Fgfr3* promoter region, which contained fourteen consensus Runx2-binding motifs, Runx2 strongly induced reporter activity (Fig. 4G). Serial deletions of the 8-kb fragment to the 2.6-kb fragment reduced Runx2-dependent transcriptional activation; however, further deletions to the 0.23-kb fragment augmented Runx2-dependent transcriptional activation. Thus, we focused on the 0.23-kb fragment, which contained two putative Runx2-binding sites, R1 and R2 (Fig. 4H). The mutation of either R1 or R2 partly reduced Runx2-dependent transcriptional activation, and the mutations of both R1 and R2 completely abolished Runx2-dependent transcriptional activation (Fig. 4I).

We then examined the binding of endogenous Runx2 in the promoter regions of *Fgfr1*, *Fgfr2*, and *Fgfr3* by ChIP assays (Fig. 4J). The promoter regions of *Fgfr1* (−88 ~+199), *Fgfr2* (−83 ~+216), and *Fgfr3* (−188 ~+178), which contained the putative Runx2-binding sites responsible for Runx2-dependent transcriptional activation, were amplified by PCR using DNA immunoprecipitated with the anti-Runx2 antibody, but not with IgG.

### Accumulation of proliferating osteoblast progenitors, which express Runx2 and Fgfr2, in calvariae and mandibles in *Sp7*^−/−^ mice, but not in *Runx2*^−/−^ mice

**Figure 5:**
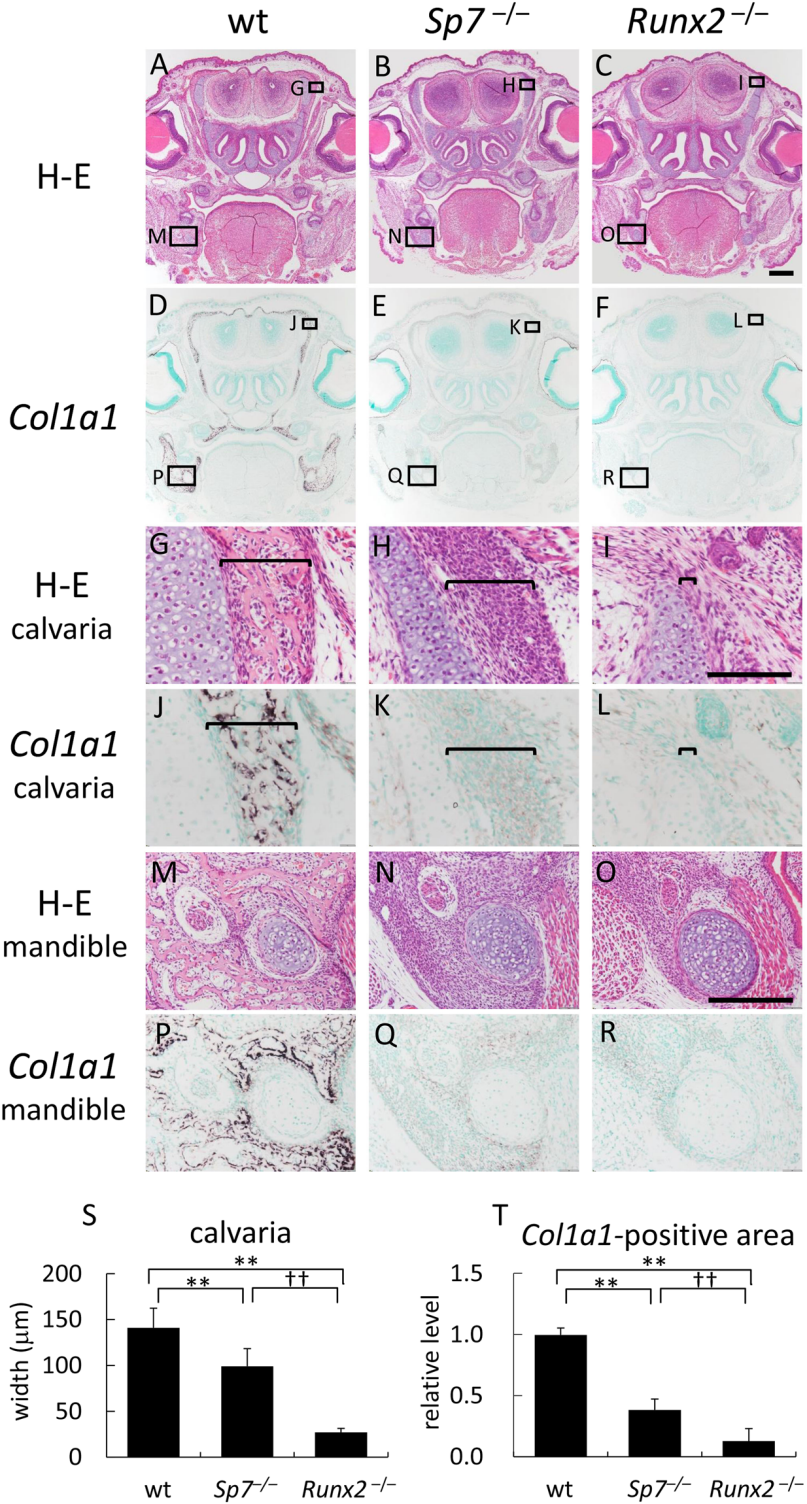
Histological analysis of *Sp7*^−/−^ and *Runx2*^−/−^ mice Frontal sections of wild-type (wt) (**A,D,G,J,M,P**), *Sp7*^−/−^ (**B,E,H,K,N,Q**), and *Runx2*^−/−^ (**C,F,I,L,O,R**) mice at E18.5 were stained with H-E (**A–C,G–I,M–O**), or subjected to *in situ* hybridization using the *Col1a1* probe (**D–F,J–L,P–R**). The boxed regions in A, B, and C are magnified in G and M, H and N, and I and O, respectively. The boxed regions in D, E, and F are magnified in J and P, K and Q, and L and R, respectively. Brackets in (G–L) indicate the layers of osteoblastic cells or osteoblast progenitors in the calvarial region. The widths of calvariae (wt: n = 8, *Sp7*^−/−^: n = 6, *Runx2*^−/−^: n = 5), and *Col1a1*-positive area in mandibles (wt: n = 4, *Sp7*^−/−^: n = 3, *Runx2*^−/−^: n = 3) were measured and shown in S and T, respectively. The values in wild-type mice were set as 1, and the relative levels are shown in T. Bars: 500 μm (**A–F**), 100 μm (**G–L**), 200 μm (**M–R**).

Although *Runx2* is expressed in mesenchymal cells in the maxilla, mandible, and perichondrium of the humerus in *Sp7*^−/−^ mice, their differentiation into osteoblasts is completely blocked^38^. Since Runx2 regulated Fgfrs in osteoblast progenitors (Figs 3 and 4), we focused on mesenchymal cells in intramembranous bone regions, including calvariae and mandibles, in *Sp7*^−/−^ mice in order to clarify the physiological roles of Fgfr gene regulation by Runx2. We compared the intramembranous bone area including the calvariae and mandibles in wild-type, *Sp7*^−/−^, and *Runx2*^−/−^ mice at E18.5 (Fig. 5). In wild-type mice, bone structures were established and osteoblasts strongly expressed *Col1a1* (Fig. 5G,J,M,P). Although *Sp7*^−/−^ mice and *Runx2*^−/−^ mice both showed no bone structure, the layer of mesenchymal cells in the calvarial region in *Sp7*^−/−^ mice was much thicker than that in *Runx2*^−/−^ mice (Fig. 5G-I,S). The expression of *Col1a1* in mesenchymal cells was weak in *Sp7*^−/−^ mice and *Runx2*^−/−^ mice, but stronger in *Sp7*^−/−^ mice than in *Runx2*^−/−^ mice, and the *Col1a1*-positive area in *Sp7*^−/−^ mandibles was much larger than that in *Runx2*^−/−^ mandibles (Fig. 5J–L,P–R,T). Therefore, mesenchymal cells, which are considered to be osteoblast progenitors, were accumulated in calvariae and mandibles of *Sp7*^−/−^ mice but not of *Runx2*^−/−^ mice.

In limb bone development in wild-type mice at E18.5, chondrocytes in epiphysis expressed *Col2a1*, and metaphysis and diaphysis were already replaced with bone and occupied by osteoblasts, which expressed *Col1a1* (Supplemental Fig. 2A,D,G,J,M,P). *Sp7*^−/−^ mice completely lacked bone formation, and the limb skeletons were cartilaginous at E18.5 (Supplemental Fig. 2B,E), as previously described^38^. Chondrocytes were maturated in the diaphysis, in which *Col2a1* expression was absent, in *Sp7*^−/−^ mice (Supplemental Fig. 2H,K). Osteoblast progenitors, which expressed *Col1a1*, were accumulated in the perichondrium of *Sp7*^−/−^ mice and some of them differentiated into morphologically chondrogenic cells, which expressed both *Col2a1* and *Col1a1* (Supplemental Fig. 2H,K,N,Q). Although the limb skeletons were also cartilaginous in *Runx2*^−/−^ mice at E18.5, chondrocyte maturation was inhibited and chondrocytes in the entire femurs expressed *Col2a1* (Supplemental Fig. 2C,F,I,L), as previously described^39,40^. The accumulation of mesenchymal cells in the perichondrium was absent, and osteoblast progenitors, which expressed *Col1a1*, were few (Supplemental Fig. 2O,R). These findings indicate that Runx2 is required for the expansion of osteoblast progenitors in the perichondrium of endochondral bones.

**Figure 6:**
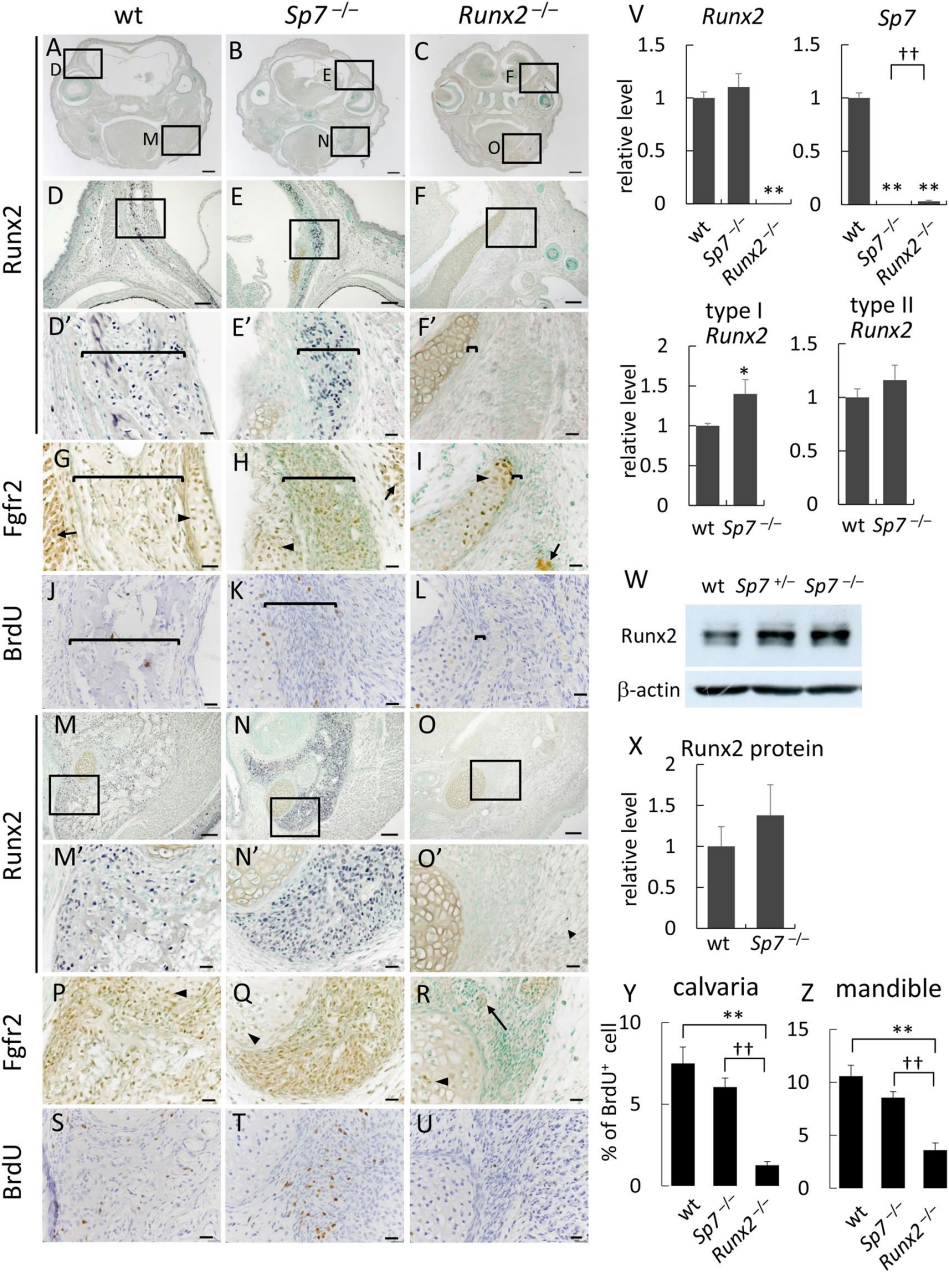
Runx2 and Fgfr2 expression and BrdU labeling in the calvaria and mandible of wild-type, *Sp7*^−/−^, and *Runx2*^−/−^ mice (**A–U**) Frontal sections of wild-type (**A**,**D**,**G**,**J**,**M**,**P**,**S**) and *Sp7*^−/−^ (**B**,**E**,**H**,**K**,**N**,**Q**,**T**), and *Runx2*^−/−^ (**C**,**F**,**I**,**L**,**O**,**R**,**U**) mice at E18.5 were reacted with the anti-Runx2 antibody (**A–F,M–O**) and anti-Fgfr2 antibody (**G**–**I**,**P**–**R**) or subjected to BrdU labeling (**J**–**L**,**S**–**U**). The boxed regions in A are magnified in D and M, the boxed regions in B are magnified in E and N, and the boxed regions in C are magnified in F and O. The boxed regions in (D–F) and (M–O) are magnified in (D’–F’) and (M’–O’), respectively. The similar regions of (D’–F’) are shown in (**G–I**) and (**J–L**), and those of (M’–O’**)** are shown in (**P–R**) and (**S–U**). Brackets in (D’–L) indicate the layers of osteoblastic cells or osteoblast progenitors in the calvarial region, short arrows in (**G–I**) indicate muscle fibers, arrowheads in (**G–I**) and (**P–R**) indicate chondrocytes, and long arrows in R indicate neurons. Bars: 500 μm (**A**–**C**), 100 μm (**D–F**,**M**–**O**), 20 μm (D’-L, M’–U). (**V**) Real-time RT-PCR analysis. RNA was extracted from the calvariae of wild-type, *Sp7*^−/−^, and *Runx2*^−/−^ mice at E18.5. The values in wild-type mice were set as 1, and relative levels are shown. Data are the mean ± SE of 4–5 mice. *vs. wild-type mice. *p < 0.05, **^,††^p < 0.01. (W) Western blot analysis. Protein was extracted from the calvariae of wild-type, *Sp7*^+/−^, and *Sp7*^−/−^ mice at E18.5. β-actin was used as an internal control. (**X**) Quantification of Western blot bands. The normalized values of Runx2 protein bands in wild-type mice were set as 1, and the relative levels in *Sp7*^−/−^ embryos are shown. Data are the mean ± SE of 3 bands. (**Y** and **Z**) BrdU-positive osteoblastic cells and osteoblast progenitors in calvariae (**Y**) and mandibles (**Z**) were counted and shown as a percentage of the number of osteoblastic cells and osteoblast progenitors. n = 6. **^,††^p < 0.01.

We then performed immunohistochemistry using the anti-Runx2 and anti-Fgfr2 antibodies to examine the expression of Runx2 and Fgfr2 in osteoblast progenitors in *Sp7*^−/−^ mice. Runx2 and Fgfr2 were strongly detected in osteoblasts in wild-type mice and osteoblast progenitors in *Sp7*^−/−^ mice, but undetectable in the mesenchymal cells in *Runx2*^−/−^ mice, although Fgfr2 was detected in chondrocytes of *Runx2*^−/−^ mice (Fig. 6A–I, M–R). Real-time RT-PCR and Western blot analyses showed that total *Runx2* mRNA, type II *Runx2* mRNA, and Runx2 protein are expressed at similar levels in wild-type and *Sp7*^−/−^ mice, that type I *Runx2* mRNA is expressed at slightly higher levels in *Sp7*^−/−^ mice than wild-type mice, and that *Sp7* mRNA levels are extremely low in *Runx2*^−/−^ mice (Fig. 6V–X).

BrdU-positive osteoblasts or osteoblast progenitors were observed at similar frequencies in wild-type and *Sp7*^−/−^ mice, whereas BrdU-positive cells were severely reduced in mesenchymal cells in the calvarial region and mandible of *Runx2*^−/−^ mice (Fig. 6J–L,S–U,Y,Z). These results indicate that Runx2 is required for the proliferation of osteoblast progenitors and the expansion of osteoblast progenitors in *Sp7*^−/−^ mice.

### Runx2 enhances the proliferation of osteoblast progenitors through Fgf signaling pathway

**Figure 7:**
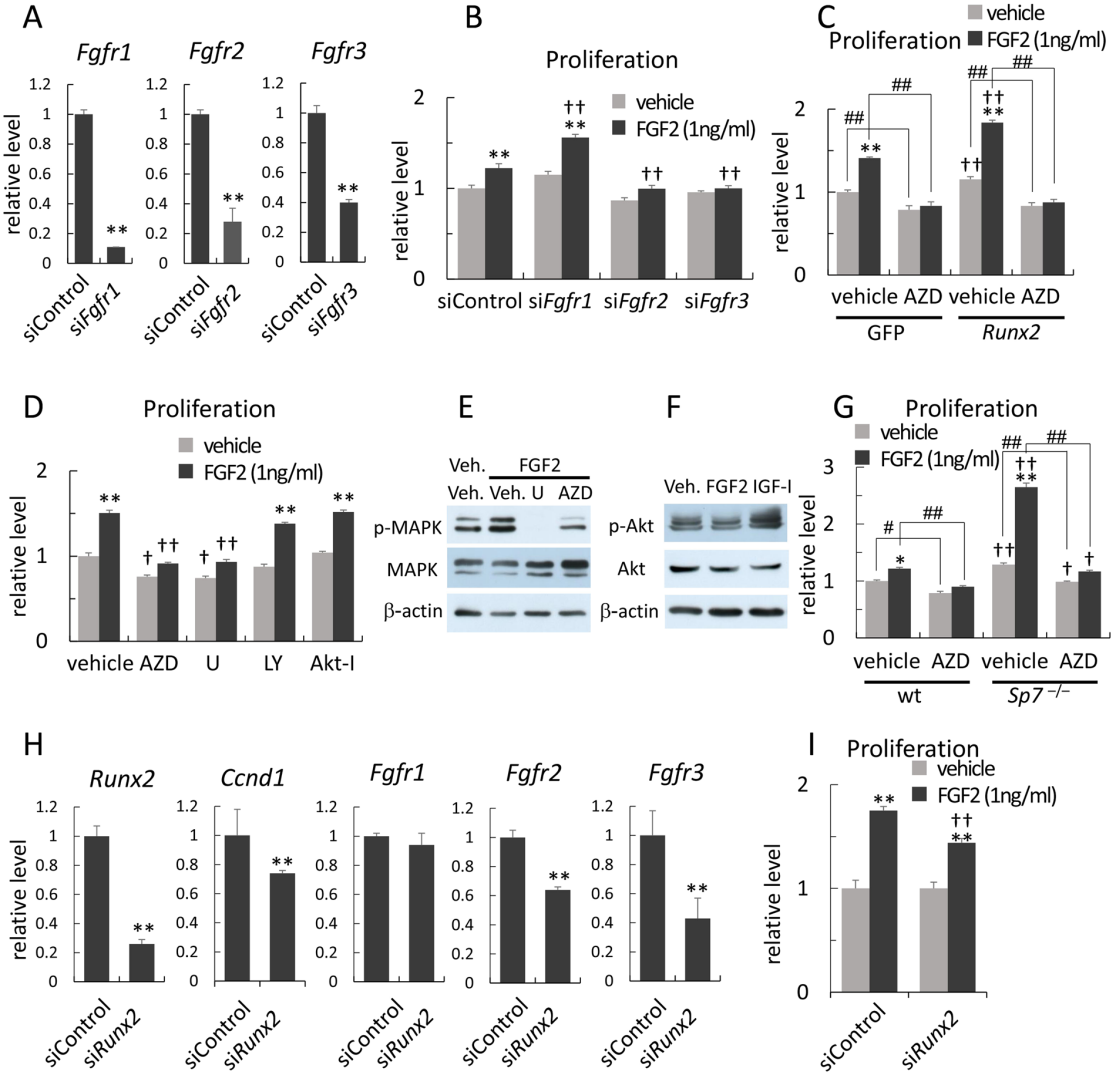
Analyses of the proliferation of osteoblast progenitors *in vitro* (**A**) Reductions in *Fgfr1*, *Fgfr2*, and *Fgfr3* mRNA by the introduction of respective siRNA into wild-type osteoblast progenitors. Each value of *Fgfr1*–*3* in the introduction of control siRNA was set as 1 and the relative levels are shown. n = 3. **p < 0.01. (**B**) Effects of FGF2 and each siRNA for *Fgfr1*, *Fgfr2*, and *Fgfr3* on the proliferation of wild-type osteoblast progenitors. The values of the vehicle in the control siRNA were set as 1, and the relative levels are shown. n = 4. * vs. the respective vehicle. ^†^vs. the respective experiment in the control siRNA. **^,††^p < 0.01. (**C**) Effects of Runx2 on proliferation and the FGF2-induced proliferation of wild-type osteoblast progenitors and the inhibition by AZD4547 (50 nM). The values of the vehicle in the GFP group were set as 1, and the relative levels are shown. n = 4. * vs. the respective vehicle. †vs. the respective experiment in the GFP group. **^,††,##^p < 0.01. (**D**) Effects of inhibitors on the FGF2-induced proliferation of wild-type osteoblast progenitors. AZD: AZD4547 (50 nM), U: U0126 (50 μM), LY: LY294002 (1 μM), Akt-I: Akt inhibitor (2.5 μM). The values in the vehicle were set as 1, and the relative levels are shown. n = 4. * vs. the respective vehicle. ^†^vs. the respective experiment in the vehicle group. ^†^p < 0.05, ^**,††^p < 0.01. (**E**,**F**) Western blots of activated p42/44 MAP kinase (**E**) and Akt (**F**). IGF-1 was used as a positive control for Akt activation (**F**). (**G**) Effects of FGF2 and AZD4547 (50 nM) on the proliferation of *Sp7*^−/−^ osteoblast progenitors. The values in the vehicle of wild-type osteoblast progenitors were set as 1, and relative levels are shown. n = 4. * vs. the respective vehicle. ^†^vs. the respective experiment in the wild-type group. *^,†,#^p < 0.05, **^,††,##^p < 0.01. (**H**) Real time RT-PCR analysis using RNA from *Sp7*^−/−^ osteoblast progenitors. The values for control siRNA were set as 1 and the relative levels are shown. n = 3. **p < 0.01. (**I**) The effects of siRNA for *Runx2* on the FGF2-induced proliferation of *Sp7*^−/−^ osteoblast progenitors. The values for the vehicle were set as 1 and the relative levels are shown. n = 4. * vs. the respective vehicle. †vs. the respective experiment in the control siRNA. **^,††^p < 0.01. Similar results were obtained in two to four independent experiments and representative data are shown in A–I.

In order to investigate the functions of Fgfr1–3 in the proliferation of osteoblast progenitors, siRNA for *Fgfr1*, *Fgfr2*, or *Fgfr3* was introduced by electroporation into wild-type osteoblast progenitors and cells were stimulated with FGF2 (Fig. 7A,B). siRNA for *Fgfr1* augmented FGF2-induced proliferation, while siRNAs for *Fgfr2* and *Fgfr3* inhibited FGF2-induced proliferation. The FGF2 treatment or the transfection of *Runx2*-expression vector by electroporation increased the proliferation of wild-type osteoblast progenitors, and the transfection of *Runx2*-expression vector enhanced FGF2-induced proliferation. The treatment with AZD4547, which is a specific Fgfr tyrosine kinase inhibitor that inhibits Fgfr1–3, reduced the proliferation of wild-type osteoblast progenitors and abrogated increases by FGF2 and/or Runx2 (Fig. 7C).

The MAPK inhibitor, U0126, exerted similar effects to AZD4547, whereas the PI3K inhibitor, LY294002, and Akt inhibitor failed to inhibit proliferation and FGF2-induced proliferation of wild-type osteoblast progenitors (Fig. 7D). In accordance with these results, the treatment with FGF2 enhanced the phosphorylation of MAPK, and this phosphorylation was strongly inhibited by U0126 and AZD, whereas the treatment with FGF2 failed to enhance the phosphorylation of Akt (Fig. 7E,F). These results indicate that Fgfr signaling through Fgfr2 and Fgfr3 regulates the proliferation of osteoblast progenitors mainly through the MAPK pathway, and that Runx2 enhances proliferation and FGF2-induced proliferation through the regulation of the Fgfr signaling pathway.

### Involvement of Runx2 in the enhancement of the proliferation of *Sp7*^−/−^ osteoblast progenitors by FGF2

The proliferation of osteoblast progenitors from *Sp7*^−/−^ calvaria was faster than wild-type osteoblast progenitors, enhanced proliferation by FGF2 was markedly stronger in *Sp7*^−/−^ osteoblast progenitors than in wild-type osteoblast progenitors, and the treatment with AZD4547 abolished accelerated proliferation and enhanced FGF2-induced proliferation (Fig. 7G). The introduction of siRNA for *Runx2* into *Sp7*^−/−^ osteoblast progenitors reduced the expression of *Ccnd1*, *Fgfr2*, and *Fgfr3* (Fig. 7H) as well as FGF2-induced proliferation (Fig. 7I).

**Figure 8:**
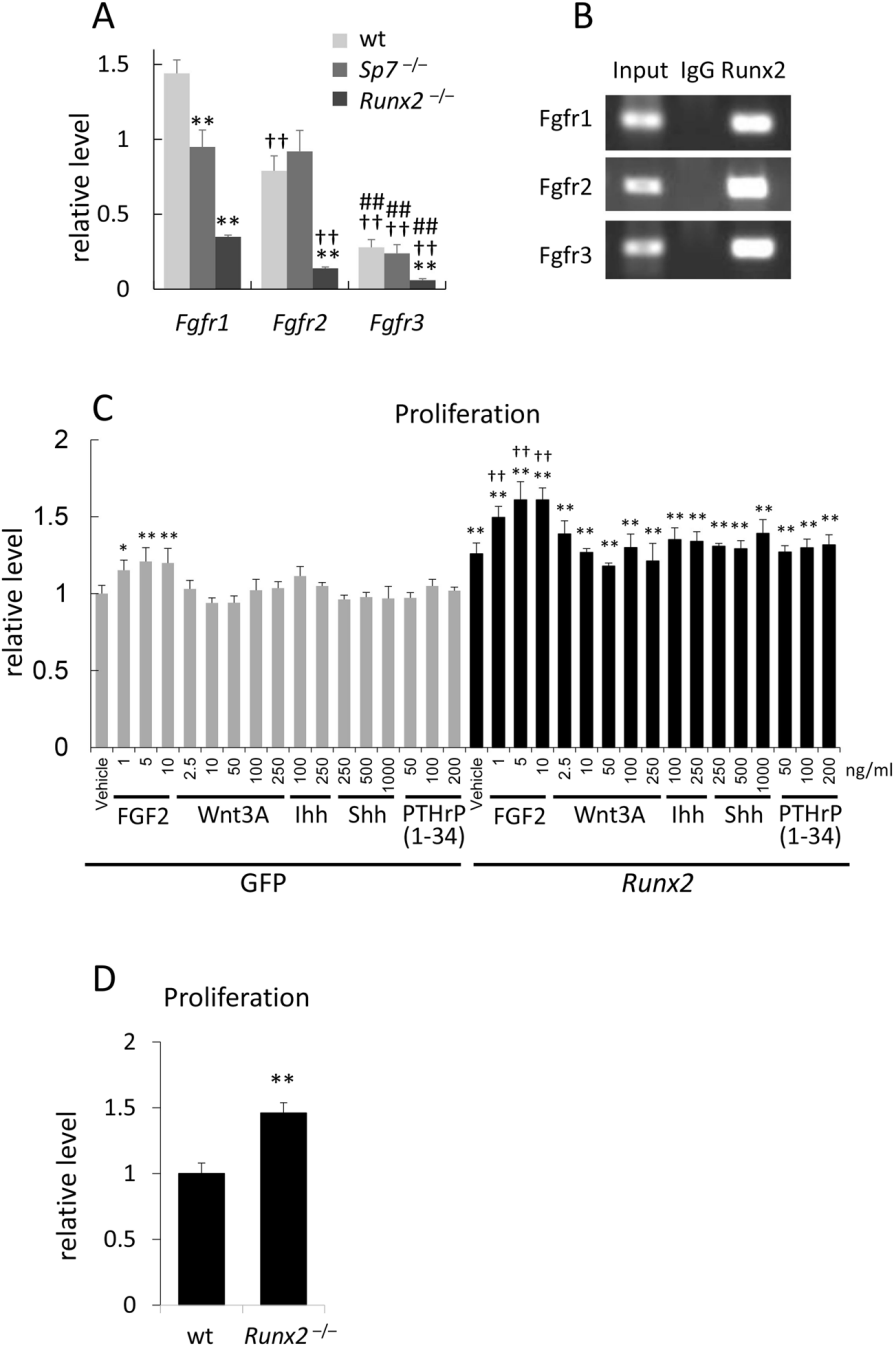
Droplet digital RT-PCR, ChIP, and cell proliferation analyses (**A**) Droplet digital RT-PCR analysis. The expression levels of *Fgfr1*–*3* were compared among wild-type, *Sp7*^−/−^, and *Runx2*^−/−^calvariae. n = 4. *vs. wild-type mice. ^†^vs. the respective mouse in *Fgfr1*. ^#^vs. the respective mouse in *Fgfr2*. **^,††,##^p < 0.01. (**B**) ChIP assay. DNA was extracted from *Sp7*^−/−^ calvariae, and DNA before (input) and after immunoprecipitation with the monoclonal anti-Runx2 antibody (Runx2) or mouse IgG (IgG) was amplified by PCR using the same primers as those in Fig. 4J. (**C**) The effects of FGF2, Wnt3a, Ihh, Shh, and PTHrP (1–34) in the proliferation of GFP- or *Runx2*-transfected wild-type osteoblast progenitors. n = 4. * vs. the vehicle in GFP-transfected cells. ^†^vs. the vehicle in *Runx2*-transfected cells. *p < 0.05, **^,††^p < 0.01. (**D**) Proliferation of wild-type and *Runx2*^−/−^ osteoblast progenitors. n = 4. **p < 0.01. Similar results were obtained in three independent experiments and representative data are shown in B-D.

We compared the expression of *Fgfr1*–*3* in the calvariae of wild-type, *Sp7*^−/−^, and *Runx2*^−/−^ mice using a droplet digital RT-PCR analysis, which detects the absolute number of each mRNA. The expression levels of *Fgfr1*–*3* were *Fgfr1* > *Fgfr2* > *Fgfr3* in wild-type and *Runx2*^−/−^ calvariae, and *Fgfr1* = *Fgfr2* > *Fgfr3* in *Sp7*^−/−^ calvariae (Fig. 8A). The expression of *Fgfr4* was undetectable in wild-type calvariae (data not shown). *Fgfr1*, *Fgfr2*, and *Fgfr3* mRNA levels were markedly lower in *Runx2*^−/−^ calvariae than in wild-type and *Sp7*^−/−^ calvariae. Although the level of *Fgfr1* was lower in *Sp7*^−/−^ calvariae than in wild-type calvariae, those of *Fgfr2* and *Fgfr3* were similar between wild-type and *Sp7*^−/−^ calvariae (Fig. 8A). In the ChIP assay using *Sp7*^−/−^ calvariae, Runx2-binding regions in *Fgfr1*, *Fgfr2*, and *Fgfr3* were amplified by PCR in DNA precipitated with the anti-Runx2 antibody, but not with IgG (Fig. 8B). These results indicate that Runx2 also directly regulates the expression of *Fgfr2* and *Fgfr3* in *Sp7*^−/−^ calvariae and enhances the FGF2-induced proliferation of *Sp7*^−/−^ osteoblast progenitors.

We also examined whether FGFs enhances the Runx2 capacity for transcriptional activation. Either FGF2 or FGF18 enhanced Runx2-dependent transcription in the reporter assays using the luciferase vector containing six tandem repeats of a Runx2 binding site (6XOSE2) (Supplemental Fig. 3).

### Signaling pathways involved in the proliferation of osteoblast progenitors

To investigate other signaling pathways than Fgf involved in the proliferation of osteoblast progenitors, the expression of cell proliferation-related genes in calvarial tissues of *Sp7*^−/−^ mice and *Runx2*^−/−^ mice was compared by microarray (Supplemental Tables 1 and 2). The expression of the genes in Wnt (*Wnt10b*, *Lef1*), hedgehog (*Ihh*), and Phtlh (*Pth1r*, *Pthlh*) signaling pathways was increased more than two times in *Sp7*^−/−^ calvarial tissues compared with *Runx2*^−/−^ calvarial tissues. Therefore, the effects of FGF2, Wnt3a, Ihh, Shh, and PTHrP (1–34) on the proliferation of wild-type osteoblast progenitors, which were transfected with either GFP- or *Runx2*-expression vector, were compared. Runx2 induced proliferation, and FGF2 but not Wnt3a, Ihh, Shh, and PTHrP (1–34) increased the proliferation in either GFP- or *Runx2*-transfected cells (Fig. 8C).

### Differential expression of the genes related to cell proliferation in *Runx2*^−/−^ osteoblast progenitors *in vitro* and *in vivo*

The proliferation of *Runx2*^−/−^ osteoblast progenitors was increased *in vitro* as previously described (Fig. 8D)^6^. To clarify the reason for the discrepancy of the proliferation capacity of *Runx2*^−/−^ osteoblast progenitors *in vitro* and *in vivo*, the expression of the cell proliferation-related genes in *Runx2*^−/−^ osteoblast progenitors was compared with that in wild-type osteoblast progenitors *in vitro*, and that in *Runx2*^−/−^ calvarial tissues was compared with that in wild-type calvarial tissues by cap analysis of gene expression (CAGE). And the genes, in which the expression ratios *in vitro* were more than two times or less than half compared with the expression ratios *in vivo*, were selected (Supplemental Tables 3 and 4). Many genes related to cell proliferation were differentially expressed in *Runx2*^−/−^ osteoblast progenitors *in vitro* and *in vivo*. Further, the expression of *Myc*, *Ccnd1*, many growth factor genes, including *Ereg*, *Hbegf*, *Tgfb1*, *Vegfa*, *Fgf7*, *Csf1*, *Fgf2*, and *Pdgfc*, and a growth factor receptor gene *Pth1r* was upregulated in *Runx2*^−/−^ osteoblast progenitors *in vitro* than *in vivo*. The differential expression of many cell proliferation-related genes *in vitro* and *in vivo* may explain why *Runx2*^−/−^ osteoblast progenitors acquired augmented capacity for proliferation *in vitro*.

### The proliferation of osteoblast progenitors in *Sp7*^−/−^ mice is dependent on the gene dosage of Runx2

**Figure 9:**
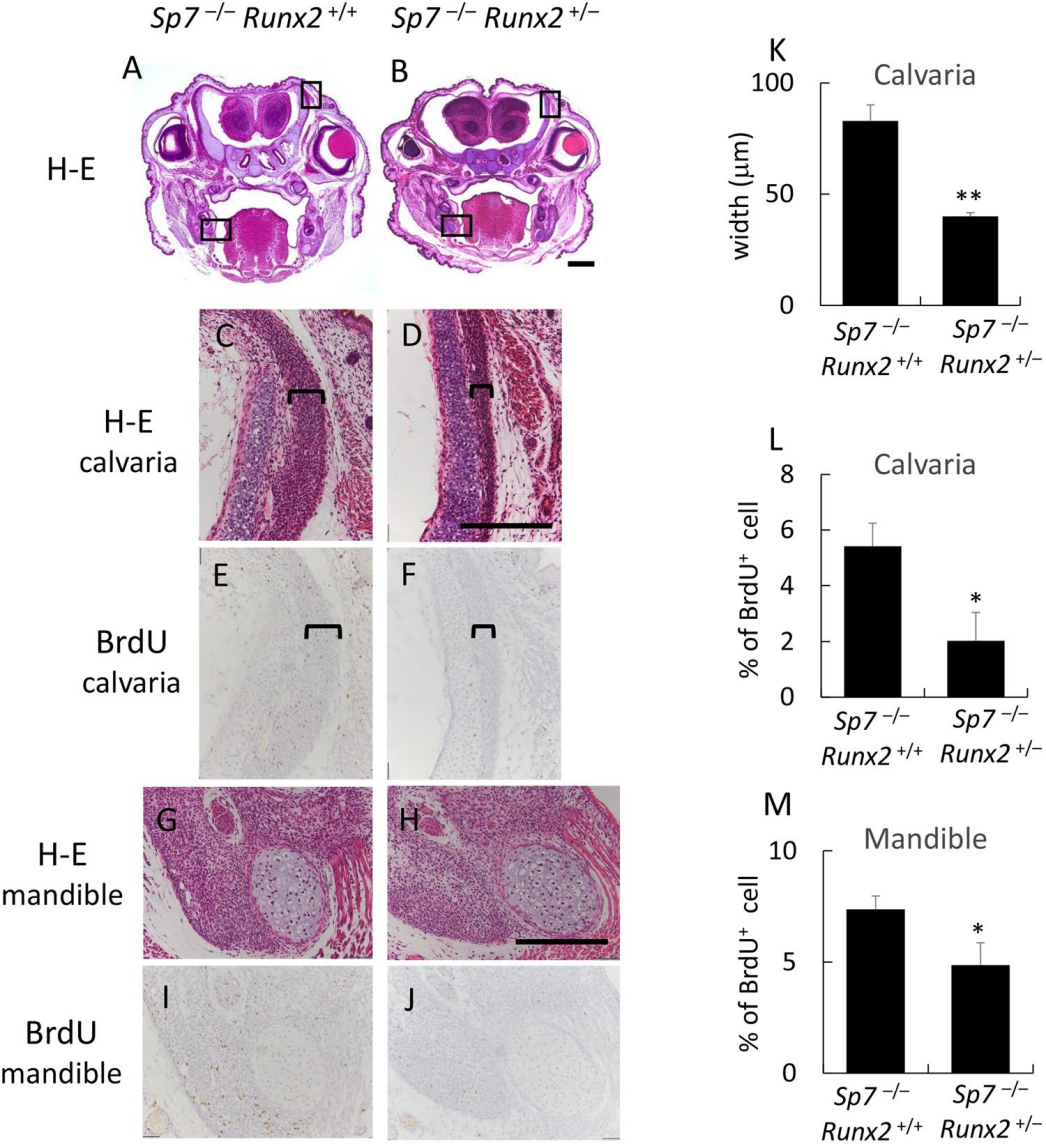
Comparison of the proliferation of osteoblast progenitors in *Sp7*^−/−^*Runx2*^+/+^ and *Sp7*^−/−^*Runx2*^+/–^ mice (**A–J**) Frontal sections of *Sp7*^−/−^*Runx2*^+/+^ (**A**,**C**,**E**,**G**,**I**) and *Sp7*^−/−^*Runx2*^+/–^ (**B**,**D**,**F**,**H**,**J**) mice at E18.5 were stained with H-E (**A**–**D**,**G**,**H**), or subjected to BrdU labeling (**E**,**F**,**I**,**J**). The upper boxed regions in A and B are magnified in C and D, and the lower boxed regions in A and B are magnified in G and H, respectively. Serial sections were used for BrdU labeling (**E,F**: calvarial region; **I,J**: mandibles). The brackets in (**C–F**) indicate the layers of osteoblast progenitors in the calvarial region. (**K**–**M**) The width of calvariae (**K**) and the percentage of BrdU-positive osteoblast progenitors in calvariae (**L**) and mandibles (**M**). The data are the mean ± SE of 3 mice. Bars: 500 μm (**A**,**B**), 200 μm (**C**–**J**).

In order to investigate whether the proliferation of osteoblast progenitors in *Sp7*^−/−^ mice is dependent on Runx2, we generated *Sp7*^−/−^*Runx2*^+/−^ mice and compared them with *Sp7*^−/−^*Runx2*^+/+^ mice (Fig. 9). The width of the layer of osteoblast progenitors in calvariae in *Sp7*^−/−^*Runx2*^+/−^ mice was about half of that in *Sp7*^−/−^*Runx2*^+/+^ mice, and the number of BrdU-positive osteoblast progenitors in calvariae was markedly lower in *Sp7*^−/−^*Runx2*^+/−^ mice than in *Sp7*^−/−^*Runx2*^+/+^ mice (Fig. 9K,L). The number of BrdU-positive osteoblast progenitors in the mandible was also lower in *Sp7*^−/−^*Runx2*^+/–^ mice than in *Sp7*^−/−^*Runx2*^+/+^ mice, but not by as much as that in calvariae (Fig. 9L,M). Interestingly, the tibiae and fibulae in *Sp7*^−/−^*Runx2*^+/+^ mice were severely bent due to the accumulation of osteoblast progenitors, whereas those in *Sp7*^−/−^*Runx2*^+/–^ mice were not bent due to the reduction in the amount of osteoblast progenitors compared with *Sp7*^−/−^*Runx2*^+/+^ mice (Supplemental Fig. 4). These results indicate that the proliferation of osteoblast progenitors in *Sp7*^−/−^ mice is dependent on the gene dosage of *Runx2*, and that the proliferation of osteoblast progenitors in calvariae is more dependent on the gene dosage of *Runx2* than that in mandibles.

## Discussion

Although *Runx2*^−/−^ mice and *Sp7*^−/−^ mice both completely lack osteoblasts and bone formation, osteoblast progenitors, which abundantly expressed Runx2, accumulated and actively proliferated in the calvaria and mandible of *Sp7*^−/−^ mice, and the number of osteoblast progenitors and their proliferation were dependent on the gene dosage of *Runx2*. Runx2 directly regulated *Fgfr1*–*3* expression, enhanced the proliferation of osteoblast progenitors, and augmented the FGF2-induced proliferation through the Fgfr2/3-MAPK signaling pathway. Further, FGF2 but not Wnt3a, Ihh, Shh, and PTHrP (1–34) increased the proliferation and augmented *Runx2*-induced proliferation. These results indicate that Runx2 is a requisite transcription factor not only for osteoblast differentiation but also for proliferation of osteoblast progenitors, and that Runx2 regulates the proliferation of osteoblast progenitors, at least partly, through the induction of *Fgfr2* and *Fgfr3* expression.

Since FGF10 binds with high affinity to Fgfr1b and Fgfr2b^41^, limb defects were likely to have been caused by the up-regulated expression of *Fgfr1b* in the mesenchyme of the limb buds of *Tg(Prrx1-EGFP-Runx2)* mice (Figs 1 and 2), which will interrupt the translocation of Fgf10 to the ectoderm leading to the failure of the induction of *Fgf8* and *Fgf4* expression in the ectoderm. Limb defects in Pfeiffer and Apert syndromes are similar to those in *Tg(Prrx1-EGFP-Runx2)* mice with low expression levels^17,23,42^. FGF2 phosphorylates Runx2 through the MAPK pathway and enhances the transcriptional activity of Runx2, ERK-dependent phosphorylation stabilizes the Runx2 protein, and Runx2 is activated through the PI3K-Akt pathway^43–46^. We also confirmed that Runx2 capacity for the transcriptional activation is enhanced by FGF2 and FGF18 (Supplemental Fig. 3). Since Runx2 directly regulated the expression of *Fgfr1*–*3*, a positive feedback loop between FGFR signaling and RUNX2 may play an important role in the pathogenesis of craniosynostosis and limb defects caused by gain-of-function mutations in *FGFR1*–*3*.

*Runx2*^−/−^ calvaria-derived osteoblast progenitors proliferated faster than wild-type osteoblast progenitors *in vitro* (Fig. 8D), as previously reported^6^. However, the number of osteoblast progenitors in calvariae and mandibles of *Runx2*^−/−^ mice was quite low, and the BrdU^+^ cells were severely reduced (Figs 5 and 6), indicating that there is a discrepancy in the proliferation of *Runx2*^−/−^ osteoblast progenitors *in vitro* and *in vivo*. It was previously reported that the expression of *Cdkn1a* (p21^CIP1^) and *Cdkn2a* (p19^ARF^) is reduced in *Runx2*^−/−^ osteoblast progenitors *in vitro*, and the introduction of Runx2 induces the expression of Cdkn1b (p27^KIP1^), *Cdkn1a*, and *Cdkn2a*, which prevent cell cycle progression through the inhibition of cyclin-dependent kinases (CDKs) or the stabilization of p53 by inhibiting Mdm2^7,47^. The report also showed that severe reduction of *Cdkn1a* and *Cdkn2a* expression in *Runx2*^−/−^ osteoblast progenitors occurs after six passages of the cells^47^. We examined the proliferation and gene expression of *Runx2*^−/−^ osteoblast progenitors after one passage of the cells, trying to mimic *in vivo* situation. *Cdkn1b* expression in *Runx2*^−/−^ osteoblast progenitors was greater than that in wild-type osteoblast progenitors, *Cdkn1a*, *Cdkn2a* (p19^ARF^), and *Cdkn2a* (p16^Ink4a^) expression in *Runx2*^−/−^ osteoblast progenitors was about 75% of wild-type osteoblast progenitors, and the introduction of *Runx2* failed to induce their expression *in vitro* (Supplemental Table 5). Further, their expression ratios in *Runx2*^−/−^ and wild-type osteoblast progenitors *in vitro* were much higher than those in *Runx2*^−/−^ and wild-type calvarial tissues *in vivo* (Supplemental Table 5), indicating that the enhanced proliferation of *Runx2*^−/−^ osteoblast progenitors *in vitro* cannot be explained by the expression levels of the CDK inhibitors. Since many genes related to cell proliferation were differentially expressed in *Runx2*^−/−^ osteoblast progenitors *in vitro* and *in vivo* (Supplemental Table 3 and 4), it seemed to be difficult to reveal the function of Runx2 in the proliferation of osteoblast progenitors by investigating *Runx2*^−/−^ osteoblast progenitors *in vitro*.

Reductions in the volume of osteoblast progenitors and their frequencies in BrdU uptake in the calvariae were greater than those in the mandibles in *Sp7*^−/−^*Runx2*^+/–^ mice relative to the respective tissues in *Sp7*^−/−^*Runx2*^+/+^ mice (Fig. 9), indicating that osteoblast progenitor proliferation is more dependent on the gene dosage of Runx2 in calvariae than mandibles. It may partly explain why open fontanelles and sutures are prominent phenotypes in cleidocranial dysplasia, which is caused by heterozygous mutation of *RUNX2*^48^. High dependency on the amount of Runx2 protein among Runx family transcription factors in calvarial bone development is also shown in the comparison of *Runx2*^+/–^ mice with conditional *Cbfb* knockout mice or *Cbfb* isoform knockout mice^49,50^. As osteoblast progenitors were scarce and the BrdU-positive cells were few in both regions of calvaria and mandible in *Runx2*^−/−^ mice (Figs 5 and 6), however, our findings also indicate that Runx2 is required for the proliferation of osteoblast progenitors in both calvaria and mandible. During endochondral bone development, osteoblast differentiation occurs first in the perichondrium, and *Sp7*-expressing preosteoblasts invade the cartilage with blood vessels and give rise to trabecular osteoblasts^51^. The accumulation of osteoblast progenitors was observed in the perichondrium of cartilaginous limb skeletons of *Sp7*^−/−^ mice but not in *Runx2*^−/−^ mice, and the accumulation of osteoblast progenitors in *Sp7*^−/−^ limb skeletons was also dependent on the gene dosage of *Runx2* (Supplemental Figs 2 and 4), indicating that Runx2 is also required for the proliferation of osteoblast progenitors for trabecular osteoblasts.

Runx2 is required for mammary gland development, and *Runx2* deletion increased animal survival in a mouse model of breast cancer with reduced proliferation and cyclin D expression^52^. The strong expression of Runx2 is associated with estrogen receptor/progesterone receptor/HER2-negative breast cancer and patients with strong Runx2 expression have a poorer survival rate than those with negative or weak expression^53^. The *FGFR2* gene has been identified as a locus associated with an increased risk of developing breast cancer, and a single nucleotide polymorphism in the *FGFR2* gene, which enhances RUNX2 binding, increases *FGFR2* expression^54–57^. Furthermore, the knockdown of *RUNX2* reduced the expression of *FGFR2* in the breast cancer cell line MCT-7^58^. Since Runx2 directly regulated *Fgfr2* and the knockdown of *Fgfr2* was effective for inhibiting the proliferation of osteoblast progenitors, the regulation of *FGFR2* by RUNX2 may also play an important role in the development and progression of some breast cancers by enhancing cell proliferation.

In conclusion, the comparison of *Sp7*^−/−^ mice, *Sp7*^−/−^*Runx2*^+/–^ mice, *and Runx2*^−/−^ mice revealed the requirement of Runx2 in the proliferation of osteoblast progenitors. Runx2 regulated it, at least partly, through the regulation of *Fgfr2* and *Fgfr3*. Since Fgf signaling enhances the ability of Runx2 for transcriptional activation, the reciprocal regulation of Runx2 and Fgf signaling will play important roles in skeletal development, the pathogenesis of craniosynostosis, and the progression of some breast cancers.

## Materials and Methods

### Generation of transgenic and gene-targeting mice

We generated *Runx2* transgenic mice under the control of the *Prrx1* promoter using enhanced green fluorescent protein (EGFP)-*Runx2* fusion DNA {*Tg(Prrx1-EGFP-Runx2)* mice}, EGFP transgenic mice under the control of the *Prrx1* promoter {*Tg(Prrx1-EGFP)* mice}, and *Runx2*^−/−^ mice as previously described^23,59^. In briefly, *Prrx1* promoter- EGFP-*Runx2* DNA fragment or *Prrx1* promoter*-*EGFP DNA fragment was injected into the pronuclei of fertilized eggs from C57BL/6 x C3H F_1_. As *Tg(Prrx1-EGFP-Runx2)* mice died at birth, we analyzed the F_0_ generation. The expression of transgenic embryos were screened by EGFP observation or real-time RT-PCR. *Sp7*^−/−^ mice were generated as previously described^60^. *Runx2*^+/–^ mice were backcrossed with C57BL/6 more than ten times. *Sp7*^+/–^ mice were generated and maintained in C56BL/6 background. Prior to the study, all experiments were reviewed and approved by the Animal Care and Use Committee of Nagasaki University Graduate School of Biomedical Sciences (No. 1403111129-21). All animal experiments were performed in accordance with the law of humane treatment and management of animals in Japan, the standards for the breeding, maintenance and reducing pains of experimental animals by Ministry of the Environment in Japan, the basic guidelines of animal experiments by Ministry of Education, Culture, Sports, Science and Technology in Japan, and the rules for animal experiments in Nagasaki University.

### Histological and immunohistochemical examinations

Tissues were fixed in 4% paraformaldehyde/0.1 M phosphate buffer and embedded in paraffin, and sections (thickness of 4 μm) were stained with hematoxylin and eosin (H-E). Immunohistochemical analyses were performed using a monoclonal anti-Runx2 antibody (1:200 dilution; Medical & Biological Laboratories, Nagoya, Japan) and polyclonal rabbit anti-Fgfr2 antibody (1:1000 dilution; GeneTex, Inc., Irvine, USA) as previously described^61^. Sections were counterstained with Methyl green. Immunohistochemistry without the anti-Runx2 antibody or anti-Fgfr2 antibody showed no significant signals (data not shown). TUNEL staining was performed using the ApopTag^®^ system (Intergen, Burlington, MA). For analysis of BrdU incorporation, we subcutaneously injected pregnant mice with 100 μg BrdU/g body weight 1 h before sacrifice. We processed the embryos for histological analysis and detected BrdU incorporation using BrdU staining kit (Invitrogen). The sections were counterstained with hematoxylin.

### *In situ* hybridization

In *in situ* hybridization, single-stranded RNA probes labeled with digoxigenin-11-UTP were prepared using a DIG RNA labeling kit (Roche, Basel Switzerland) according to the manufacturer’s instructions. Sections were hybridized using mouse *Fgf8* and *Col1a1* antisense probes as described previously^39^. Whole mount *in situ* hybridization was performed using *Fgf10*, *Fgf8*, *Fgf4*, and *Shh* antisense probes as described previously^62^. The *in situ* hybridization of sections and whole embryos using sense probes showed no significant signals (data not shown).

### Real-time RT-PCR

Real-time RT-PCR was performed using a THUNDERBIRD SYBR qPCR Mix (Toyobo) and Light Cycler 480 real-time PCR system (Roche Diagnostics). TaqMan PCR for *Fgfr1*, *Fgfr2*, *Fgfr3*, *Runx1*, *Runx2*, *Runx3*, and *Sp7* was performed using a THUNDERBIRD Probe qPCR Mix (Toyobo). Primer sequences and information on TaqMan Probes are shown in Supplemental Table 6. We normalized values to that of *β-actin*.

### Cell culture and adenoviral transfer

Wild-type, *Sp7*^−/−^, and *Runx2*^−/−^ osteoblast progenitors were prepared from calvariae at E18.5. The calvariae were cut into small pieces and cultured for 10–14 days in three-dimensional collagen gel (Cell matrix, Nitta Gelatin, Co., Osaka, Japan) with α-modified Minimum (α-MEM) containing 10% FBS. The cells outgrowing from the explants were retrieved by incubation for 30 min with 0.2% collagenase (Wako Pure Chemical Industries, Osaka, Japan) in PBS(−) at 37 °C. In this method, the main cell types isolated were osteoblast progenitors and osteoblasts at an early differentiation stage with low alkaline phosphatase activity and virtually no osteocalcin production^59^. Cells were plated in 24-well plates at a density of 5 × 10^4^/well in αMEM supplemented with 10% fetal bovine serum (FBS). At confluency, cells were infected with an adenovirus expressing EGFP or type II *Runx2*-EGFP at a multiplicity of infection of 10 for 2 hrs. The mouse pluripotent mesenchymal cell line, C3H10T1/2, was purchased from the RIKEN Cell Bank (Tsukuba, Japan), and cultured in BME supplemented with 10% FBS.

### Reporter Assays

A 10-kb fragment of the *Fgfr1* promoter region was subcloned into the firefly luciferase reporter vector pGL4.10Luc2 (Promega, Madison, WI) from the BAC clone. A 2.5-kb fragment of the *Fgfr2* promoter region and 8-kb fragment of the *Fgfr3* promoter region were amplified by PCR using mouse genomic DNA and cloned into pGL4.10Luc2. All truncated constructs were prepared using the restriction enzyme sites or by PCR amplification. C3H10T1/2 cells were transfected with plasmid DNAs (each luciferase reporter vector 0.1 μg; pRL-Tk Renilla 0.1 μg; pSG5 or pSG5-Runx2 0.05 μg) using FuGENE 6 Transfection Reagent (Roche). Luciferase activities were examined by using Dual-Luciferase Reporter Assay System (Promega), and normalized to Renilla luciferase activity.

### ChIP assay

Chromatin immunoprecipitation (ChIP) was performed with a Chromatin Immunoprecipitation Assay Kit (Upstate Biotechnology, Billerica, MA) using the anti-Runx2 monoclonal antibody in Fig. 4J (Medical & Biological Laboratories), anti-Runx2 antibody in Fig. 8B (Santa Cruz Biotechnology, Santa Cruz, CA), or mouse IgG (Cell Signaling, Danvers, MA), using primers in Supplemental Table 6.

### Western blot analysis

A Western blot analysis was performed using anti-Runx2 (Cell Signaling), rabbit anti-phospho-p44/42 MAPK (Thr202/Tyr204) (Cell Signaling), rabbit anti-phospho-Akt (ser473) (Cell Signaling), rabbit anti-p44/42 MAPK (Cell Signaling), rabbit anti-Akt (Cell Signaling), and anti-β-actin (Santa Cruz Biotechnology) antibodies.

### Cell proliferation assay

A total of 5 × 10^5^ cells were subjected to electroporation with 1.0 μg of either the EGFP or type II *Runx2* expression vector or with 10 pmol of siRNA for *Fgfr1*, *Fgfr2*, *Fgfr3* (Bonac, Kurume, Japan), or *Runx2* (Thermo scientific, Waltham, MA) using the Neon Transfection System (Invitrogen) and cultured on 100-mm dishes for 24 h, and then the transfected cells were seeded at 1 × 10^4^ cells/well on 96-well plates. After 6 hrs, FGF2 (PeproTech Inc. Rocky Hill NJ), Wnt3a (R&D Systems, Minneapolis, MN), Ihh (R&D Systems), Shh (R&D Systems), or PTHrP(1–34) (PeproTech Inc.) was added. After 48 hrs, cell numbers were counted using Cell Counting Kit-8 (DOJINDO, Kumamoto, Japan). Inhibitors (AZD4547: Abcom, Cambridge, UK; U0126: Wako Pure Chemical Industries, Osaka, Japan; LY294002: Merck Calbiochem, Darmstadt, Germany; Akt inhibitor: Merck Calbiochem) were added 1 h before the addition of FGF2.

### Droplet digital PCR

The absolute quantity of mRNA was measured by the QX200 Droplet Digital PCR System (BIO-RAD) using EvaGreen application reagents (BIO-RAD). The absolute values of mRNA were normalized to those of β-actin mRNA.

### Statistical analysis

Data are described as the mean ± SEM, if not specified. Statistical analyses were performed using an analysis of variance followed by the Tukey-Kramer test. P < 0.05 was considered to be significant.

## Electronic supplementary material

Supplementary Information
