# Supplementary material for: Runx2 is required for the proliferation of osteoblast progenitors and induces proliferation by regulating Fgfr2 and Fgfr3
Source: Sci Rep. 2018 Sep 10;8:13551. doi: 10.1038/s41598-018-31853-0 (PMC6131145; doi:10.1038/s41598-018-31853-0)

Runx2 is required for the proliferation of osteoblast progenitors and induces proliferation by regulating *Fgfr2* and *Fgfr3*

Tetsuya Kawane[1], Xin Qin[1], Qing Jiang[1, 2], Toshihiro Miyazaki[1], Hisato Komori[1], Carolina Andrea Yoshida[1], Viviane Keiko dos Santos Matsuura-Kawata[1], Chiharu Sakane[1], Yuki Matsuo[1], Kazuhiro Nagai[3], Takafumi Maeno[1,4], Yuki Date[1, 5], Riko Nishimura[6], and Toshihisa Komori[1, 2*]

[1]Department of Cell Biology, [5]Department of Molecular Bone Biology, Unit of Basic Medical Sciences, [2]Basic and Translational Research Center for Hard Tissue Disease, Nagasaki University Graduate School of Biomedical Sciences, Nagasaki 852-8588, Japan

[3]Transfusion and Cell Therapy Unit, Nagasaki University Hospital, Nagasaki 852-8501, Japan

[4]Department of Orthopedic Surgery, Osaka City University Graduate School of Medicine, Osaka 545-8585, Japan

[6]Department of Molecular and Cellular Biochemistry, Osaka University of graduate School of Dentistry, Osaka 565-0871, Japan

*Corresponding author:

Department of Cell Biology, Unit of Basic Medical Sciences, Nagasaki University Graduate School of Biomedical Sciences, 1-7-1 Sakamoto, Nagasaki 852-8588, Japan

TEL: +81-95-819-7630; FAX: +81-95-819-7633

E-mail: komorit@nagasaki-u.ac.jp

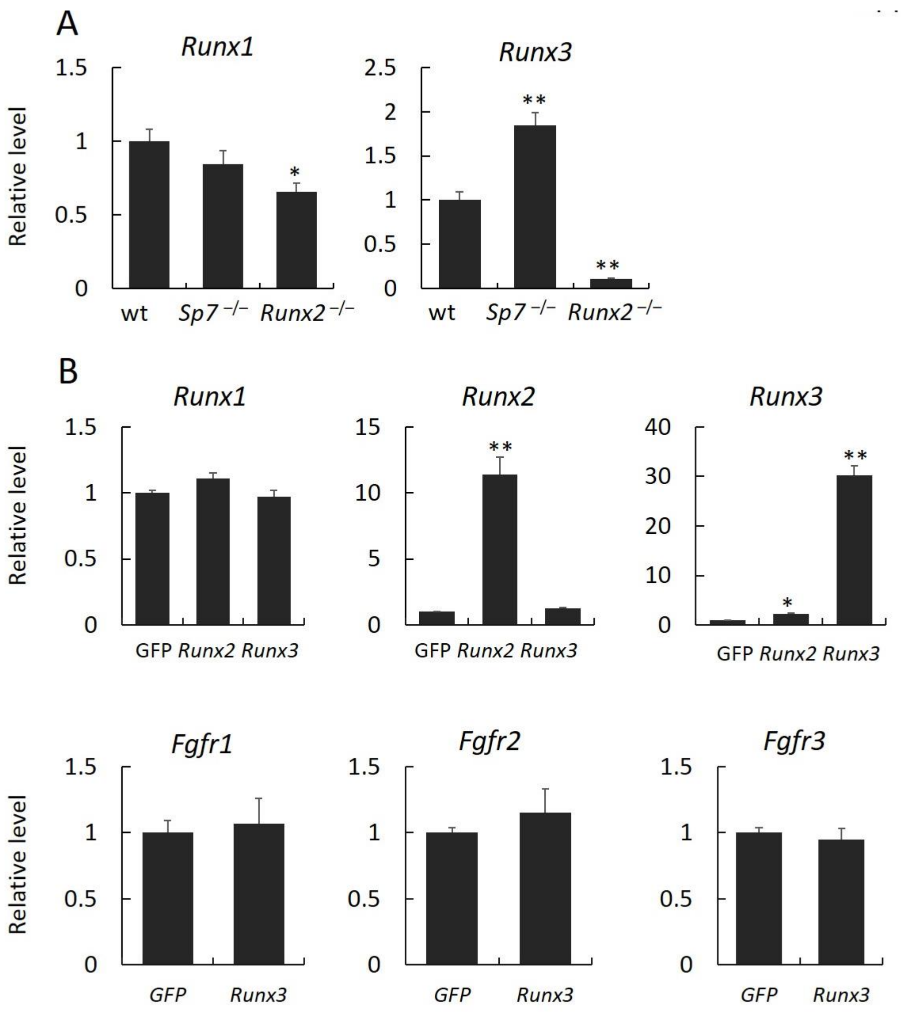

Supplemental Figure 1

Real-time RT-PCR analysis

(A) *Runx1* and *Runx3* mRNA expression in the calvariae of wild-type, *Sp7*$^{-/-}$, and *Runx2*$^{-/-}$ mice. Data are the mean ± SE of 4-5 mice. * vs. wild-type mice. *p<0.05, **p<0.01. (B) Effects of the overexpression of *Runx2* or *Runx3* on the mRNA expression of *Runx1*, *Runx2*, *Runx3, Fgfr1*, *Fgfr2,* and *Fgfr3* in osteoblast progenitors. n=3. * vs. GFP. *p<0.05, **p<0.01. Similar results were obtained in two independent experiments and representative data are shown.

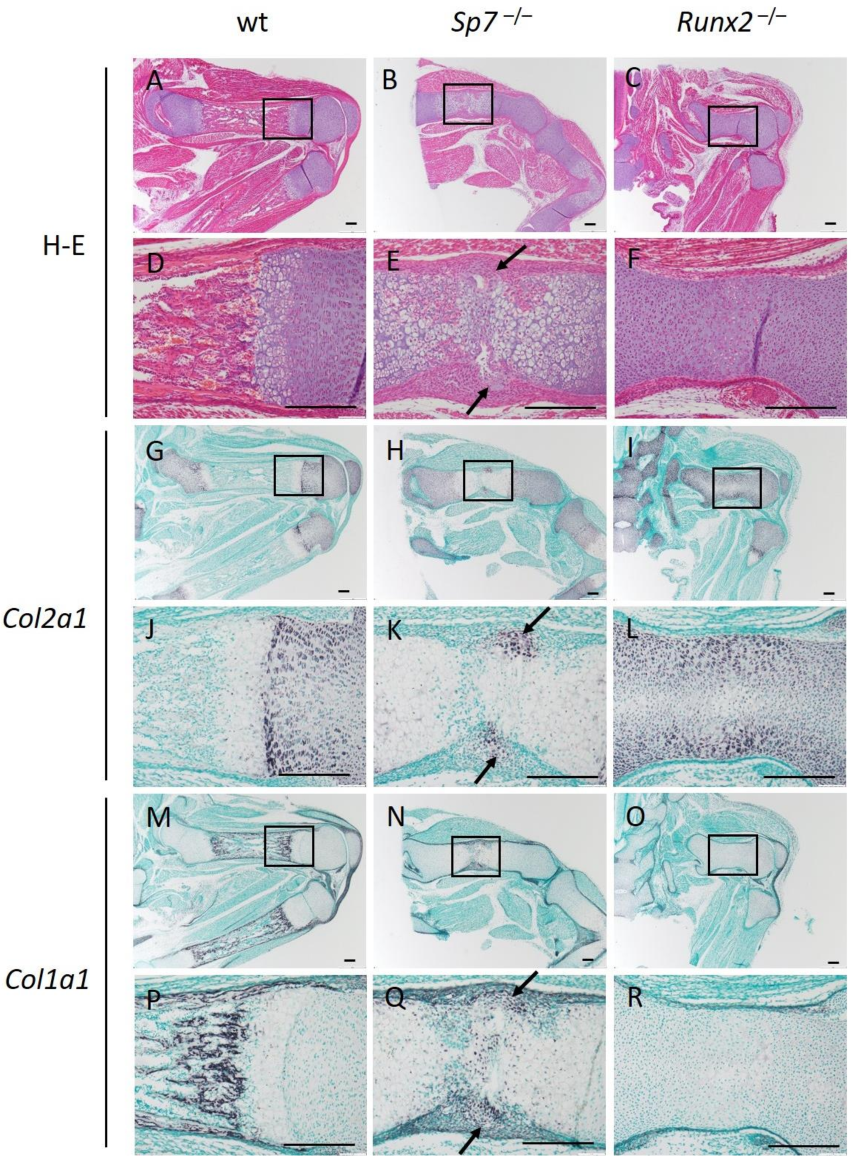

Supplemental Figure 2

Osteoblast progenitors in the perichondrium of hind limb bones in wild-type, *Sp7*$^{-/-}$, and *Runx2*$^{-/-}$ mice at E18.5

H-E stained sections (A-F) and in situ hybridization using *Col2a1* (G-L) and *Col1a1* (M-R) probes in wild-type (A, D, G, J, M, P), *Sp7*$^{-/-}$ (B, E, H, K, N, Q), and *Runx2*$^{-/-}$ (C, F, I, L, O, R) mice. The boxed regions in A-C, G-I, and M-O are magnified in D-F, J-L, and P-R, respectively. Arrows indicate chondrogenic cells, which expressed both *Col2a1* and *Col1a1*. Bars: 200 μm.

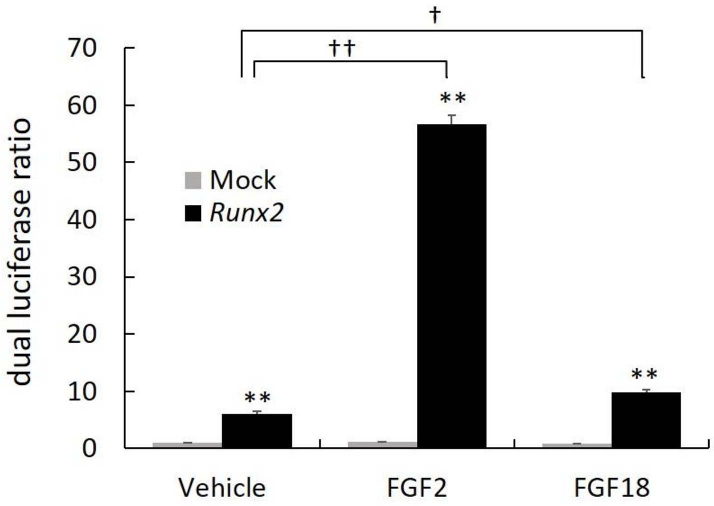

Supplemental Figure 3

Reporter assay.

The activation of a p6xOSE2 reporter vector by *Runx2* was assayed in the presence of FGF2 (10 ng/ml) or FGF18 (10 ng/ml) in *Runx2*$^{-/-}$ calvaria-derived cells line (CA120-4) [*1]. * vs. Mock. **, ††p<0.01.

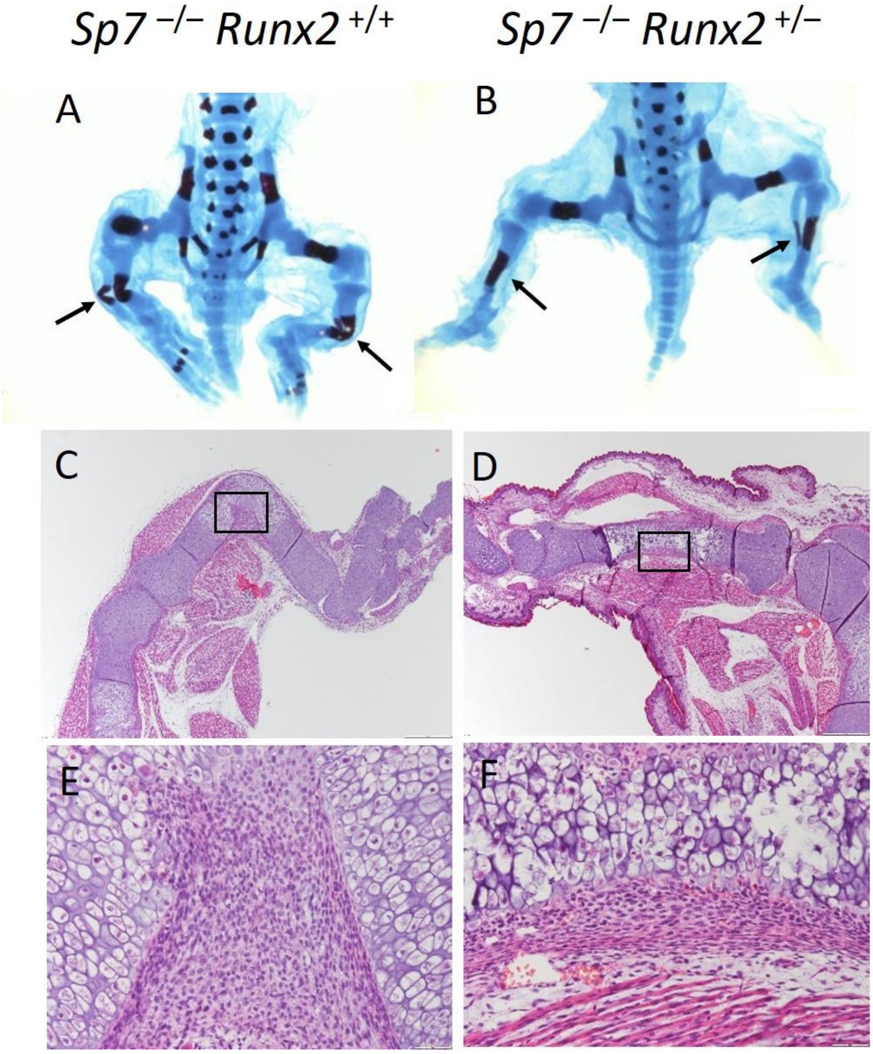

Supplemental Figure 4

Skeletal preparation and histological analysis of limb skeletons in *Sp7*$^{-/-}$*Runx2*$^{+/+}$ and *Sp7*$^{-/-}$*Runx2*$^{+/-}$ mice at E18.5

(A, B) Skeletal preparation of *Sp7*$^{-/-}$*Runx2*$^{+/+}$ (A) and *Sp7*$^{-/-}$*Runx2*$^{+/-}$ (B) mice. (C-F) H-E stained sections of *Sp7*$^{-/-}$*Runx2*$^{+/+}$ (C, E) and *Sp7*$^{-/-}$*Runx2*$^{+/-}$ (D, F) mice. The boxed regions in C and D are magnified in E and F, respectively. Arrows in A and B show tibiae and fibulae.

Supplemental table 1

Cell proliferation related-genes up-regulated more than two times in calvarial tissues of *Sp7*$^{-/-}$ mice compared with those of *Runx2*$^{-/-}$ mice at E18.5 by microarray analysis

	Gene	*Sp7*$^{-/-}$/ *Runx2*$^{-/-}$		Gene	*Sp7*$^{-/-}$/ *Runx2*$^{-/-}$
1	Bcl11b	12.97	26	Fgf1	2.65
2	Cxcl10	10.81	27	Fezf2	2.45
3	Alox12	9.24	28	Pthlh	2.43
4	Wnt10b	9.08	29	Nr2e3	2.42
5	Tff2	5.66	30	Il24	2.40
6	Nccrp1	5.35	31	Bcl6	2.39
7	Ghrh	5.16	32	Bnipl	2.38
8	Atf3	5.05	33	Fgfbp1	2.32
9	Ihh	4.76	34	Trp53inp1	2.28
10	Dynap	4.52	35	Epo	2.26
11	Alox12	4.16	36	Gata3	2.20
12	Ptgs2	4.04	37	Slfn1	2.20
13	Hck	4.02	38	Lef1	2.19
14	Edn3	3.71	39	Adarb1	2.17
15	Myocd	3.62	40	Il6	2.17
16	Tfap2a	3.61	41	Ptges	2.16
17	Bcl11b	3.55	42	Chrna7	2.13
18	Cdkn2b	3.30	43	Klf13	2.11
19	Nox1	3.27	44	Lta	2.06
20	Bmp7	3.25	45	H19	2.04
21	Vdr	3.23	46	Agt	2.03
22	Pth1r	2.93	47	Axin2	2.02
23	Drd2	2.93	48	Il3	2.02
24	Pkp2	2.76	49	Fgfr3	2.02
25	Hrg	2.68	50	Ifit3	2.02

Supplemental table 2

Cell proliferation related-genes down-regulated to less than half in calvarial tissues of *Sp7*$^{-/-}$ mice compared with those of *Runx2*$^{-/-}$ mice at E18.5 by microarray analysis

	Gene	*Sp7*$^{-/-}$/ *Runx2*$^{-/-}$		Gene	*Sp7*$^{-/-}$/ *Runx2*$^{-/-}$
1	Pla2g2a	0.20	27	Fgf10	0.38
2	Plau	0.24	28	Thpo	0.38
3	Plac8	0.24	29	Meg3	0.39
4	Calcrl	0.28	30	Smad1	0.39
5	Adrb2	0.29	31	Areg	0.39
6	Ptk2	0.29	32	Prox1	0.41
7	Gnrhr	0.29	33	Edn1	0.41
8	Meg3	0.29	34	Pim2	0.42
9	Pou3f2	0.29	35	Wnt2	0.42
10	Ntn1	0.30	36	Fzd5	0.42
11	Tbx5	0.31	37	Fabp4	0.42
12	Chp2	0.31	38	Xirp1	0.43
13	Cntfr	0.31	39	Ptk2b	0.44
14	Zbtb16	0.32	40	Hif1a	0.45
15	Ptprv	0.33	41	Lbx1	0.45
16	Nodal	0.34	42	Notch1	0.46
17	Mas1	0.35	43	T	0.47
18	Il1rl1	0.35	44	Cxcl12	0.47
19	Tgfbr1	0.35	45	Sfrp2	0.47
20	Tdgf1	0.36	46	Isl1	0.47
21	Mas1	0.37	47	Meg3	0.47
22	Nacc2	0.37	48	Hras1	0.47
23	Osm	0.37	49	Il34	0.48
24	Dpt	0.37	50	Asph	0.48
25	Gpc3	0.38	51	Cxcl12	0.49
26	Hoxa3	0.38	52	Col18a1	0.49
			53	Wt1	0.50

Supplemental table 3

The ratios of mRNA expression by CAGE in *Runx2*$^{-/-}$ and wild-type osteoblast progenitors in vitro (a), the ratios of mRNA expression by CAGE in *Runx2*$^{-/-}$ and wild-type calvarial tissues (b), and their ratios (a/b).
The genes, in which a/b were more than two, are shown. The genes with more than 10 counts per million in *Runx2*$^{-/-}$ osteoblast progenitors in vitro were analyzed.

		a *Runx2*$^{-/-}$ /wt (*in vitro*)	b *Runx2*$^{-/-}$ /wt (*in vivo*)	a/b			a *Runx2*$^{-/-}$ /wt (*in vitro*)	b *Runx2*$^{-/-}$ /wt (*in vivo*)	a/b
1	Il11	3.88	0.01	457.43	38	Dkc1	0.88	0.27	3.19
2	Fosl1	0.86	0.02	57.37	39	Fgf7	0.76	0.24	3.16
3	Timp1	1.16	0.02	47.58	40	Tead4	1.19	0.39	3.07
4	Ereg	1.00	0.03	33.04	41	Bcl6	1.14	0.37	3.07
5	Ptgs2	0.88	0.04	22.36	42	Hipk1	1.55	0.51	3.04
6	Inhba	0.44	0.02	21.56	43	Csf1	0.72	0.24	2.99
7	Hmga2	1.41	0.07	20.85	44	Col18a1	2.15	0.73	2.96
8	Cdkn2b	0.58	0.04	16.33	45	Cd63-ps	0.94	0.33	2.88
9	Crlf1	1.45	0.09	15.91	46	Tpd52	1.83	0.64	2.84
10	Hbegf	0.81	0.05	14.94	47	Spry2	1.16	0.42	2.79
11	Ptges	1.08	0.08	13.99	48	Itpripl2	1.06	0.40	2.63
12	Itpripl2	2.63	0.31	8.61	49	Tbx3	2.34	0.90	2.59
13	Tgfb1	1.05	0.14	7.58	50	Fgf2	1.78	0.70	2.54
14	Osmr	1.05	0.14	7.36	51	Clcf1	1.11	0.44	2.50
15	Ngf	1.02	0.15	6.91	52	Eef1e1	0.70	0.28	2.49
16	Plau	1.04	0.17	6.22	53	Ets1	0.99	0.41	2.44
17	Hmox1	0.67	0.11	6.01	54	Ndufaf4	0.99	0.41	2.43
18	Il1rl1	0.45	0.08	5.94	55	Tnk2	1.87	0.78	2.38
19	Serpine2	1.48	0.26	5.73	56	S1pr1	0.62	0.26	2.38
20	Slc1a5	1.04	0.19	5.41	57	Yars	0.83	0.35	2.36
21	Vegfa	0.94	0.19	5.01	58	Bax	0.78	0.33	2.36
22	Cdkn1a	0.76	0.16	4.82	59	Pdgfc	0.61	0.26	2.33
23	Btg1	1.65	0.36	4.53	60	Rpl23	1.06	0.46	2.31
24	Pdpn	1.25	0.29	4.31	61	Dot1l	0.80	0.35	2.29
25	Pth1r	0.74	0.17	4.28	62	Atf5	0.82	0.36	2.28
26	Spred3	1.58	0.37	4.27	63	Aimp2	0.75	0.34	2.20
27	Eif5a	1.15	0.28	4.12	64	Cth	0.78	0.36	2.18
28	Suv39h2	1.43	0.35	4.11	65	Acer3	1.23	0.57	2.16
29	Has2	1.12	0.28	4.00	66	Ptprf	1.36	0.64	2.12
30	Cd9	1.14	0.29	3.99	67	Rps4y2	0.95	0.45	2.11
31	Ifitm3	1.90	0.49	3.84	68	Jun	0.81	0.39	2.10
32	Shmt2	0.91	0.24	3.78	69	Dlg1	1.38	0.66	2.08
33	Myc	0.75	0.20	3.70	70	Rps9	1.35	0.65	2.07
34	Fam129b	1.19	0.32	3.66	71	Smyd2	0.99	0.48	2.06
35	Gtpbp4	0.94	0.27	3.51	72	Smad1	1.11	0.54	2.06
36	Adm	0.72	0.21	3.41	73	Zfp703	1.02	0.50	2.02
37	Ccnd1	0.89	0.26	3.40	74	Rbpj	1.06	0.53	2.00

Supplemental table 4

The ratios of mRNA expression by CAGE in *Runx2*$^{-/-}$ and wild-type osteoblast progenitors in vitro (a), the ratios of mRNA expression by CAGE in *Runx2*$^{-/-}$ and wild-type calvarial tissues (b), and their ratios (a/b).

The genes, in which a/b were less than 0.5, are shown. The genes with more than 10 counts per million in *Runx2*$^{-/-}$ osteoblast progenitors in vitro were analyzed.

		a *Runx2*$^{-/-}$ /wt (*in vitro*)	b *Runx2*$^{-/-}$ /wt (*in vivo*)	a/b
1	Fgfr1op	0.00	0.01	0.00
2	Igf1	0.14	7.42	0.02
3	Sfrp1	0.52	10.25	0.05
4	Wnt9a	0.26	3.75	0.07
5	Itga1	0.31	3.92	0.08
6	Clec11a	0.07	0.70	0.09
7	Scx	0.73	7.24	0.10
8	Cxcl12	0.79	7.38	0.11
9	Gpc3	0.29	2.20	0.13
10	Odz3	0.79	5.19	0.15
11	Csnk2a1	0.29	1.50	0.20
12	Cd248	1.42	7.16	0.20
13	Wdr6	0.62	2.98	0.21
14	Slit3	0.24	1.12	0.22
15	Id4	0.50	2.29	0.22
16	Figf	2.06	8.97	0.23
17	Egfr	0.70	3.05	0.23
18	Igf2	0.31	1.31	0.24
19	Nfib	0.93	3.51	0.26
20	Dab2ip	0.68	2.52	0.27
21	Ptn	0.58	2.08	0.28
22	Il11ra1	0.88	2.93	0.30
23	Slc9a3r1	1.08	3.58	0.30
24	Ddr1	0.91	2.99	0.30
25	Ccnd3	0.59	1.91	0.31
26	Rogdi	0.90	2.84	0.32
27	Efemp1	1.30	4.09	0.32
28	Zfp503	0.60	1.89	0.32
29	Kifap3	0.73	2.10	0.35
30	Cav1	0.64	1.84	0.35
31	Podn	0.81	2.25	0.36
32	Wnt5a	1.02	2.80	0.36
33	Insr	0.44	1.16	0.38
34	Cyp1b1	0.81	2.14	0.38
35	Brip1	0.79	2.04	0.39
36	Lrrcc1	0.80	2.04	0.39
37	Gm3511	1.04	2.59	0.40
38	Tob1	0.80	2.00	0.40
39	Pttg1	0.76	1.88	0.40
40	Gja1	0.52	1.26	0.41
41	Vps13b	0.58	1.42	0.41
42	Prkaca	1.06	2.60	0.41
43	Ptprk	0.72	1.69	0.43
44	Tgfb2	0.55	1.26	0.44
45	Sox11	0.83	1.83	0.45
46	Efnb2	0.71	1.51	0.47
47	Tsc22d1	0.49	1.04	0.47
48	Gnas	0.95	1.97	0.48
49	Kdm4c	0.76	1.59	0.48
50	Cbx8	0.98	1.98	0.49
51	Tgfbr2	0.80	1.62	0.50

Supplemental table 5

Comparison of the mRNA expression of Cdk inhibitors by CAGE

The values of *Runx2*$^{-/-}$ osteoblast progenitors with GFP transfection, *Runx2*$^{-/-}$ osteoblast progenitors with *Runx2* transfection, wild-type osteoblast progenitors with GFP transfection, wild-type osteoblast progenitors with *Runx2* transfection, *Runx2*$^{-/-}$ calvarial tissues, and wild-type calvarial tissues are shown. The ratios of *Runx2*$^{-/-}$ and wild-type osteoblast progenitors with GFP transfection, and the ratios of *Runx2*$^{-/-}$ and wild-type calvarial tissues are also shown.

	cell culture					calvarial tissue		
	Runx2$^{-/-}$ (*GFP*)	*Runx2*$^{-/-}$ (*Runx2*)	wt (*GFP*)	wt (*Runx2*)	*Runx2*$^{-/-}$ (*GFP*)/ wt (*GFP*)	*Runx2*$^{-/-}$	wt	*Runx2*$^{-/-}$ /wt
Cdkn1a ($p21^{Cip1}$)	250.89	269.74	331.74	362.30	0.76	28.68	182.72	0.16
Cdkn1b ($p27^{Kip1}$)	69.17	66.43	47.59	51.09	1.45	62.29	75.60	0.82
Cdkn1c ($p57^{Kip2}$)	1.03	5.24	0.88	7.08	1.17	496.90	79.44	6.26
Cdkn2a ($p16^{Ink4a}$)	18.10	16.29	24.48	25.43	0.74	0.00	0.00	–
Cdkn2a ($p19^{ARF}$)	10.99	11.78	14.49	18.39	0.76	0.00	1.48	0.00
Cdkn2b ($p15^{Ink4b}$)	21.41	27.49	37.11	46.67	0.58	0.40	11.32	0.04
Cdkn2c ($p18^{Ink4c}$)	17.80	18.43	11.60	12.74	1.53	9.09	8.04	1.13
Cdkn2d ($p19^{Ink4d}$)	0.00	0.00	1.37	0.00	0.00	0.00	0.00	–
Cdkn3 (KAP)	11.71	14.89	8.81	8.87	1.33	6.05	6.68	0.91

Supplemental Table 6

Sequences of the oligonucleotides for real-time RT-PCR and ChIP analyses.

	gene	organism	direction	5'->3'
Real-time PCR	*Runx2*	mouse	F	TCCACCACGCCGCTGTCT
			R	TCAGTGAGGGATGAAATGCT
Real-time PCR	*Sp7*	mouse	F	AGGCACAAAGAAGCCATAC
			R	AATGAGTGAGGGAAGGGT
Real-time PCR	*Fgfr1*	mouse	F	GCTCCCTACTGGACATCC
			R	TGCGGAGATCGTTCCACG
Real-time PCR	*Fgfr2*	mouse	F	GTCTCCGAGTATGAGTTG
			R	ACTATGACGTAGAGAGGT
Real-time PCR	*Fgfr3*	mouse	F	CCAGAAACGTCCTGGTGA
			R	ACTGGGATGCCAGGATAC
Real-time PCR	*Fgfr1b*	mouse	F	AATTAATAGCTCGGATGC
			R	TGCCACAGGTCTGGTGACAG
Real-time PCR	*Fgfr1c*	mouse	F	ATACCACCGACAAGGAAAT
			R	AGAGTTACCCGCCAAGCACG
Real-time PCR	*Fgfr2b*	mouse	F	GGATAAATAGCTCCAATGCA
			R	GCGCTTGCTGTTTGGGCA
Real-time PCR	*Fgfr2c*	mouse	F	GGTGTTAACACCACGGACAA
			R	TGCAGAGTGAAAGGATAT
Real-time PCR	*Fgfr3b*	mouse	F	AGTCCTGGATCAGTGAGAATGT
			R	GTGAACACGCAGCCAAAAG
Real-time PCR	*Fgfr3c*	mouse	F	TAACACCACCGACAAGGAGC
			R	AGCCACGCAGAGTGATGGGA
Real-time PCR	Type I *Runx2*	mouse	F	CGTTGCAGCCACCGCCAGG
			R	CAAGTTCGAGGAAGCCGTGTGC
Real-time PCR	Type II *Runx2*	mouse	F	CCAGCCACCGAGACCAACC
			R	GTTTGACGCCATAGTCCCTCC
Real-time PCR	*Ccnd1*	mouse	F	GGCACCTGGATTGTTCTGTT
			R	CAGCTTGCTAGGGAACTTGG
Real-time PCR	*β-actin*	mouse	F	CCACCCGCGAGCACAGCTTC
			R	TTGTCGACGACCAGCGCAGC
ChIP primer	*Fgfr1*	mouse	F	GCTGCAGCTTGAGAACTACAAGG
			R	AGGTCGCCGCAATGCGCTAA
ChIP primer	*Fgfr2*	mouse	F	AGGAAACGGCTCGGGTTTCA
			R	TGCCTACTCCAAACTTTGCT
ChIP primer	*Fgfr3*	mouse	F	TATTGGAGGGAGAAGCGT
			R	TTTTCCAGGGTCTCCTCT
TaqMan PCR	*Runx1*	mouse		Mm00486762_m1 (Applied Biosystems)
TaqMan PCR	*Runx3*	mouse		Mm00490666_m1 (Applied Biosystems)
TaqMan PCR	*Fgfr1*	mouse		Mm00438930_m1 (Applied Biosystems)
TaqMan PCR	*Fgfr2*	mouse		Mm01269930_m1 (Applied Biosystems)
TaqMan PCR	*Fgfr3*	mouse		Mm00433294_m1 (Applied Biosystems)
TaqMan PCR	*β-actin*	mouse		Mm00607939_s1 (Applied Biosystems)

Supplementary method

Analysis of mRNA expression using cDNA arrays

Total RNA was extracted from E18.5 calvariae using the acid guanidine thiocyanate-phenol-chloroform method according to the manufacturer's instruction (Isogen, Nippon Gene, Tokyo, Japan). Poly(A) mRNA was purified from total RNA using an Oligotex kit (Takara, Tokyo, Japan). The cRNA was amplified, labeled and hybridized to an Agilent SurePrint G3 Mouse Gene Expression Microarray 8 x 60 K (Agilent Technologies, Santa Clara, CA) according to the manufacturer’s instructions. The hybridized microarray slides were scanned by means of an Agilent scanner. Relative hybridization intensities and background hybridization values were calculated using Agilent Feature Extraction Software (ver. 9.5.1.1), and background hybridization values were calculated using Agilent Feature Extraction Software (ver. 9.5.1.1).

CAGE library preparation and sequencing

For CAGE analysis, $5x10^5$ cells were subjected to electroporation with 1 μg of each expression vector using the Neon Transfection System (Invitrogen) and cultured on 24-well plates for 24h. Total RNA was extracted using ISOGEN (NipponGene) from cultured cells or calvarial tissue. A multiplexed CAGE library was constructed from 2 - 5 μg total RNA sample based on nAnT-iCAGE protocol [*2] (K. K. DNAForm, Yokohama, Japan). Briefly, total RNA was reverse transcribed using a random “N6 plus base 3” primer (TCTNNNNNN), using Superscript III reverse transcriptase (Thermo Fisher). Following oxidation (with sodium peroxide) and biotinylation of the m7G cap structures, first-strand-complete messenger RNA: complementary DNA (mRNA:cDNA) hybrids were bound with streptavidin beads, pulled down with a

magnet, and released. This was followed by RNaseH and RNaseONE treatments, ligation of the 5′ linker, and ligation of 3′ linker comprising 6 nt index sequence. Finally, second-strand synthesis was performed, creating the final double-stranded DNA product. Multiplex deep sequencing of cDNA libraries was performed on an Illumina NextSeq500 sequencer. The sequenced CAGE tags were mapped to mouse reference genomes, mm9, using the BWA software without changing any parameters. Unmapped reads are then mapped to mm9 using HiSAT2. For tag clustering, the CAGE-tag 5’-coordinates were input for CAGEr clustering followed by differential expression analysis using R program DESeq2.

*1 Enomoto H, Shiojiri S, Hoshi K, Furuichi T, Fukuyama R, Yoshida CA *et al*. Induction of osteoclast differentiation by Runx2 through receptor activator of nuclear factor-kappa B ligand (RANKL) and osteoprotegerin regulation and partial rescue of osteoclastogenesis in *Runx2*$^{-/-}$ mice by RANKL transgene. *J Biol Chem*. 2003; **278**: 23971-23977

*2 Murata M, Nishiyori-Sueki H, Kojima-Ishiyama M, Carninci P, Hayashizaki Y, Itoh M. Detecting expressed genes using CAGE. *Methods Mol Biol.* 2014; **1164**: 67-85.

Supplementary Information

Fig.4

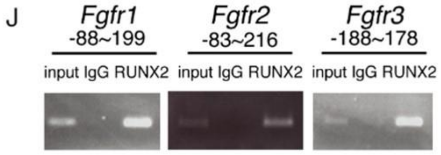

Original gels:

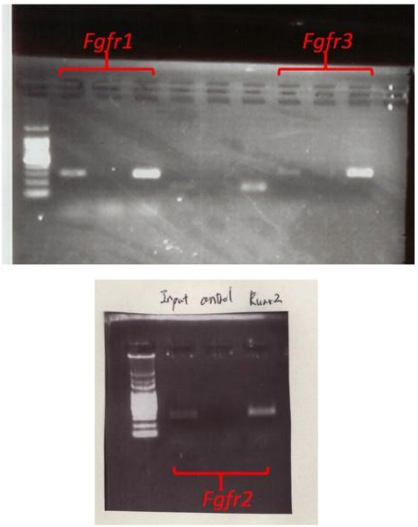

Fig.6

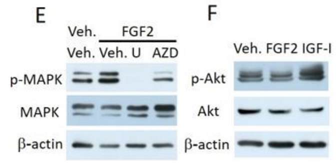

Original films:

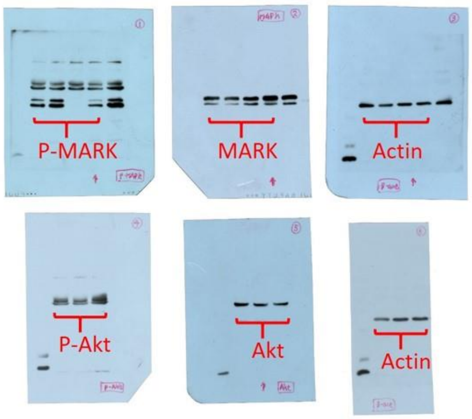

Fig.7

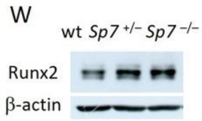

Original films:

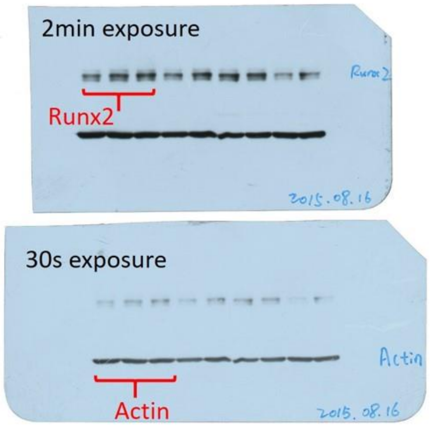

Fig.8

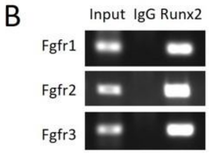

Original gels:

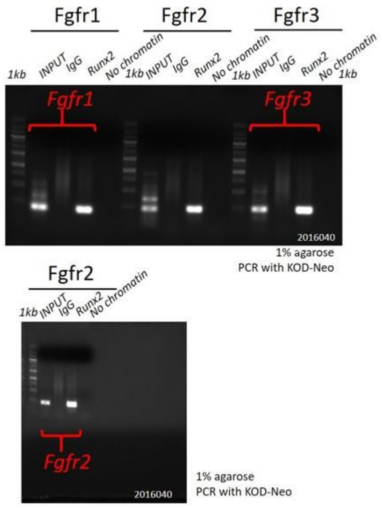

Supplement: Supplementary file 1 — Supplementary Information [file 41598_2018_31853_MOESM1_ESM.pdf]
